# Design, synthesis, antimicrobial, antibiofilm evaluation and *Z*/*E*-isomerization of novel 6-((arylamino)methylene)benzo[*a*] phenazin-5(6*H*)-ones induced by organic solvent †

**DOI:** 10.1039/d3ra05788g

**Published:** 2023-10-09

**Authors:** Abolfazl Olyaei, Nooshin Ghaleghovandi, Foozieh Moghadami, Mahdieh Sadeghpour, Shohreh Abediha

**Affiliations:** a Department of Chemistry, Payame Noor University (PNU) PO BOX 19395-4697 Tehran Iran olyaei_a@pnu.ac.ir; b Department of Biology, Payame Noor University Tehran Iran; c Department of Chemistry, Islamic Azad University Qazvin Branch Qazvin Iran

## Abstract

New 6-((arylamino)methylene)benzo[*a*]phenazin-5(6*H*)-one derivatives were synthesized, and good-to-high yields were achieved through one-pot, four-component condensation of 2-hydroxy-1,4-naphthoquinone, 1,2-phenylenediamine, aromatic amines and triethyl orthoformate using formic acid as catalyst under solvent-free conditions at 90 °C. The structure of these new compounds was confirmed using FT-IR and ^1^H-NMR as well as MS spectroscopy. Investigation of spectroscopy data indicated that the synthesized compounds exist in the keto-enamine tautomeric form and undergo *Z*/*E*-isomerization around the C=C bond in DMSO-*d*_6_ at room temperature. Furthermore, intramolecular hydrogen bond has been observed in the synthesized *E*- and *Z*-ketoenamines. The noticeable features of the present procedure availability of starting materials, very simple operation, easy work-up, short reaction times, good to high yields and no need for column chromatography separation of benzophenazine enamines. The newly synthesized compounds were evaluated *in vitro* for their antibacterial, antifungal and antibiofilm activities against some of the tested microorganisms. The results demonstrated that compound 6b showed the maximum antibacterial activity, 6d exhibited the maximum antifungal activity and 6b had the most efficiency to inhibit biofilm formation of *Bacillus subtilis* (80%) at 200 μg mL^−1^ concentration.

## Abstract 2

New 6-((arylamino)methylene)benzo[*a*]phenazin-5(6*H*)-one derivatives were synthesized, and good yields were achieved through one-pot condensation of 2-hydroxy-1,4-naphthoquinone, 1,2-phenylenediamine, aromatic amines and triethyl orthoformate using formic acid under solvent-free conditions at 90 °C.

## Graphical abstract

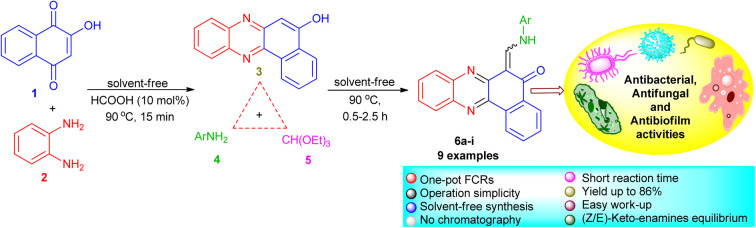

## Introduction

Phenazine compounds are nitrogen-containing heterocycles that exist in natural and synthetic products. They have been demonstrated various pharmaceutical activities including antimalarial,^1^ antiphrastic,^1^ antiplatelet,^2^ trypanocidal,^3^ fungicidal,^4^ antitumor,^5^ and antimicrobial.^6^ Among phenazins, the synthesis of benzo[*a*]phenazines^7^ has been given special attention due to their unique biological properties in various fields such as antioxidants,^8^ anti-tumor,^9^ anti-AIDS,^10^ and treatment of Alzheimer's disease.^11,12^ Enaminones are also known as β-enamino ketones, are a class of useful building blocks in synthetic heterocyclic compounds.^13,14^ In addition, enaminone itself is also a substructure that frequently occurred in natural products and biologically functional compounds.^15,16^ Therefore, it is not surprising that the research on enaminone synthesis has also become a topic of broad attention. The term enaminones usually refers to the compounds that contain the conjugate system O=C–C=C–N which comprises three electrophilic centers and two nucleophilic centers.^17^ Several methods for the preparation of enaminones involves reaction between ammonia or primary or secondary amine and 1,3-diketone or 3-keto ester. A straightforward route is provided by the condensation of 1,3-dicarbonyl compounds with amines^18^ and by the Mannich reaction of CH(OEt)_3_, ketone, and amine.^19^ In recent years, several novel methods for the synthesis of enaminones have been developed.^20^ Enaminones have a pronounced tendency to undergo various isomeric transformations in solvents with different polarities. This property is particularly important for applications of enaminones and plays a major role in determining chemical, biological, and therapeutic activities of these compounds. According to the ^1^H- and ^13^C-NMR spectroscopy, they have feature intramolecular hydrogen bonds of O–H⋯N and N–H⋯O type, tautomerism between the enol-imine and keto-enamine forms, and *Z*/*E*-isomerization of the keto-enamine form in respect to the C=C bond.^21^ In continuation of our interests in synthesis of enol-imine and keto-enamine compounds by one-pot, multi-component reactions,^22–26^ herein, we report the one-pot, four-component synthesis of a new series of 6-((arylamino)methylene)benzo[*a*]phenazin-5(6*H*)-one derivatives by condensation reaction of 2-hydroxy-1,4-naphthoquinone, 1,2-phenylenediamine, aromatic amines and triethyl orthoformate using formic acid as catalyst under solvent-free conditions and evaluation of antibacterial, antifungal and antibiofilm activities of target compounds against some of the tested microorganisms.

## Result and discussions

To obtain the optimal reaction conditions for the synthesis of benzophenazine enamines, the reaction of 2-hydroxy-1,4-naphthoquinone (1, 1.0 mmol), 1,2-phenylenediamine (2, 1.0 mmol), 2-amino-6-methylpyridine (4a, 1.0 mmol), and triethyl orthoformate (5, 1.0 mmol) was chosen as a model reaction. Initially, to minimize by-product formation, lawsone and *o*-phenylenediamine were condensed under solvent-free conditions in the presence of HCOOH (10 mol%) at 90 °C to form an orange solid of benzo[*a*]phenazin-5-ol (3)^27^ after approximately 15 min. Then, EtOH (5 mL), 2-amino-6-methylpyridine (4a), and triethyl orthoformate (5) were added to the reaction mixture. The mixture was stirred at room temperature and the progress of the reaction was followed by thin-layer chromatography (TLC), which was resulted the formation of the product 6a in 10% yield after 5 h (Table 1, entry 1). To improve the yield of 6a, the reaction was carried out in EtOH under reflux conditions, but a small increase in yield was exhibited (Table 1, entry 2). After the above investigation, we also tested the significance of other solvents such as H_2_O, EtOH/H_2_O (1 : 1), MeOH, CH_3_CN and glac.HOAc under reflux conditions, but no gratifying results were attained (Table 1, entries 3–7). Then, the reaction was carried out under solvent-free conditions at 70, 90 and 120 °C (Table 1, entries 8–10). The best result was obtained in terms of yield (83%) and reaction time (0.5 h) when the reaction was performed at 90 °C under solvent-free conditions (Table 1, entry 9). This series of experiments reveal that the optimal results were obtained when the reaction was conducted at 90 °C under solvent-free conditions.

**Table 1:** Optimization of the reaction conditions 6a^a^

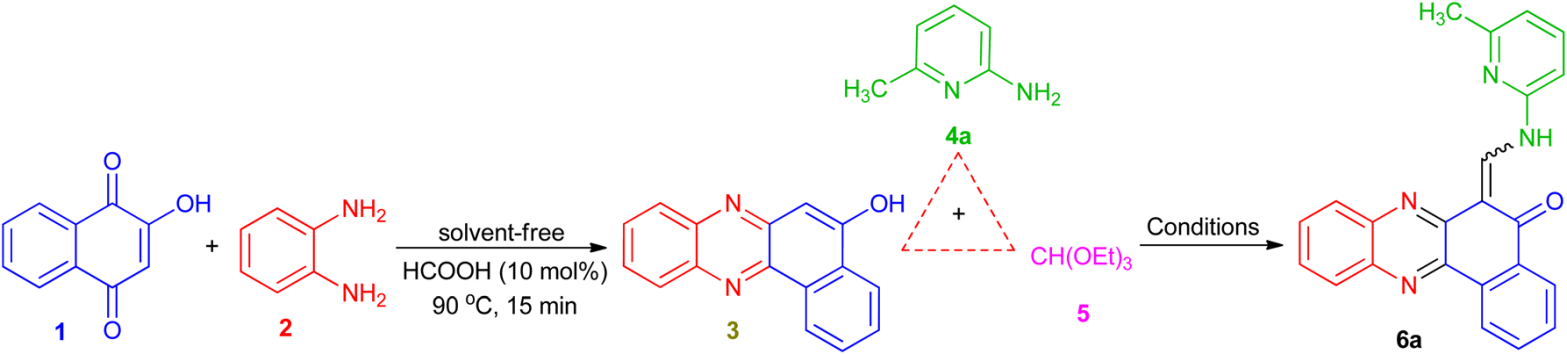				
Entry	Solvent	Temperature (°C)	Time (h)	Yield^b^ (%)
1	EtOH	r.t.	5	10
2	EtOH	Reflux	2.5	18
3	H_2_O	Reflux	3	44
4	EtOH:H_2_O	Reflux	2.5	25
5	CH_3_OH	Reflux	3	48
6	CH_3_CN	Reflux	1.5	33
7	glac.HOAc	Reflux	3.5	18
8	—	70	1	56
9	—	90	0.5	83
10	—	120	0.5	64

a Reagents and conditions: lawsone (1, 1.0 mmol), 1,2-phenylenediamine (2, 1.0 mmol), 2-amino-6-methylpyridine (4a, 1.0 mmol), triethylortoformate (5, 1.0 mmol), HCOOH (10 mol%), and solvent (10 mL).

b Isolated yields.

These optimized reaction conditions were then used to synthesize and explore the scope of this novel transformation with various aryl and heteroaryl amines containing electron-donating and electron-withdrawing groups such as 2-amino-3-methylpyridine, 2-amino-4-methylpyridine, 3-aminopyridine, 4-aminopyridine, 2-aminopyrimidine, 4-chloroaniline, 4-methylaniline and 4-methyoxyaniline, to give a series of 6-((arylamino)methylene)benzo[*a*]phenazin-5(6*H*)-one derivatives 6a–i (Table 2). All reactions proceeded efficiently, and the desired products were isolated pure in good to high yields by the addition of CH_3_CN to the reaction mixture followed by filtration. As can be seen from Table 2, electronic effects and the nature of substituent on the aryl amines resulted in products with different reaction times and yields. When the heteroaryl amines such as aminopyridines were employed under the optimized reaction conditions, the reactions proceeded smoothly with lower reaction times and afforded the corresponding products in high yields. In addition, when the anilines with electron-donating and electron-withdrawing groups like 4-Me, 4-OMe and 4-Cl were employed the reactions gave the desired products in good yields. As a result, heteroaryl amines showed higher reactivity and gave better yields than aniline derivatives.

**Table 2:** Synthesis of 6-((arylamino)methylene)benzo[*a*]phenazin-5(6*H*)-one derivatives 6a–i

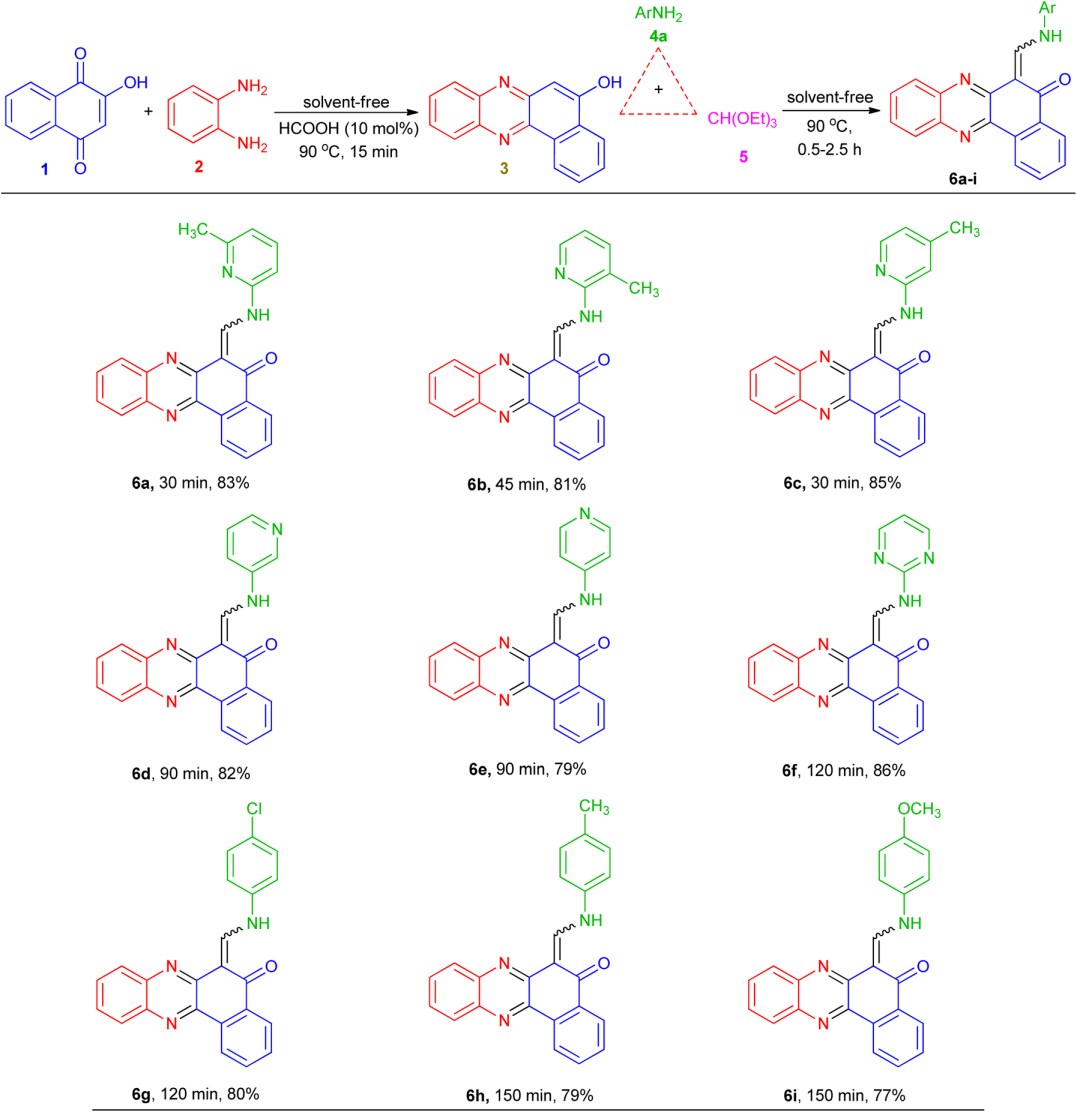

**Fig. 1:**
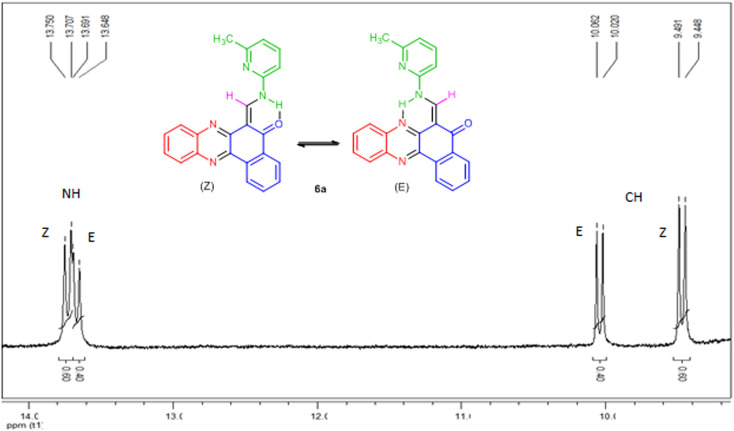
^1^H-NMR spectrum of compound 6a.

All the synthesized compounds were unknown to the best of our knowledge and were characterized by FT-IR, ^1^H-NMR, mass spectroscopy and melting points. The FT-IR spectra of the target products showed the absorptions at *ῡ* = 1650–1690 cm^−1^ due to carbonyl group. The ^1^H-NMR spectra indicated that all of the synthesized compounds 6a–i existed in DMSO-*d*_6_ solution as a mixture of *E*- and *Z*-keto-enamine isomers. For instance, the ^1^H-NMR spectrum of compound 6a consisted of two downfield doublet signals for the NH group at *δ* = 13.73 and 13.67 ppm. As indicated in Fig. 1, the more downfield signal corresponds to the *Z*-isomer of compound 6a which is stabilized by a strong chelate type intramolecular hydrogen bond with the oxygen of the C=O group. The signal for the proton of the NH group in the more upfield region corresponds to the *E*-isomer. The formation of another intramolecular hydrogen bond with nitrogen atom of the C=N fragment is possible in this isomer which is significantly weaker than the hydrogen bond between the NH and C=O groups. With such an assignment the integral intensities of the NH signals allow quantitative determination of the corresponding isomer content in the equilibrium mixtures: the *Z*-isomer from the signal at 13.73 ppm (60%) and the *E*-isomer from the signal at 13.67 ppm (40%). The methylene proton was discernible as two doublets at *δ* = 9.47 and 10.04 ppm for *Z* and *E* isomers, respectively. The difference between the CH proton chemical shift values for the two geometric isomers is substantially smaller, and the CH signal from the *Z*-isomer is located upfield. It is interesting to note the difference between the values of the vicinal spin–spin coupling constants of the CH and NH protons for the two isomeric forms are 12.9 Hz, indicating a transoid arrangement of the N–H and CH bonds. The aromatic protons resonated in the region *δ* = 7.09–8.91 ppm. It should be noted that there is no signal of hydroxyimine tautomer in the ^1^H-NMR spectra of the synthesized compounds. Moreover, the NH signals disappear upon addition of D_2_O into the NMR sample, and the protons of CH moiety collapse into a singlet. In the IR spectrum of compound 6a, the peaks at 3396, 1650 cm^−1^ are related to the stretching frequencies of N–H and C=O, respectively. Mass spectra of 6a–i reveal the presence of the molecular ion peaks and other fragments consistent with the assigned structures. Chemical shifts (*δ*) of enaminic protons, ^*3*^*J*_HH_ and *E*/*Z* isomers ratio of 6a–i are listed in Table 3. It should be noted that despite of heating the samples in DMSO-*d*_6_ prior to ^13^C-NMR measurement, the low solubility of such compounds makes it difficult to receive a clean ^13^C-NMR spectrum.

**Table 3:** Chemical shifts (*δ*) of enaminic protons and *E*/*Z* isomers ratio of 6a–i in DMSO-*d*_6_

Compd.	*δ* NH (ppm)		*δ* CH (ppm)		^*3*^ *J*_HH_ (Hz)		*E*/*Z* (%)	
	*E*-isomer	*Z*-isomer	*E*-isomer	Z-isomer	*E*-isomer	*Z*-isomer	*E*-isomer	*Z*-isomer
6a	13.67	13.73	10.04	9.47	12.9	12.9	40	60
6b	13.36	13.90	9.21	9.83	12.3	12.0	70	30
6c	13.59	13.67	10.03	9.47	12.6	12.6	40	60
6d	13.75	13.84	9.49	8.96	13.2	13.2	35	65
6e	13.38	13.60	9.43	8.89	12.9	12.9	32	68
6f	12.43	12.52	9.09	9.26	13.2	13.2	42	58
6g	12.85	13.79	9.40	9.19	13.2	12.9	30	70
6h	13.86	13.93	8.92	9.46	13.5	13.5	40	60
6i	13.84	13.98	9.34	8.82	13.2	13.2	40	60

### The proposed reaction mechanism

**Scheme 1:**
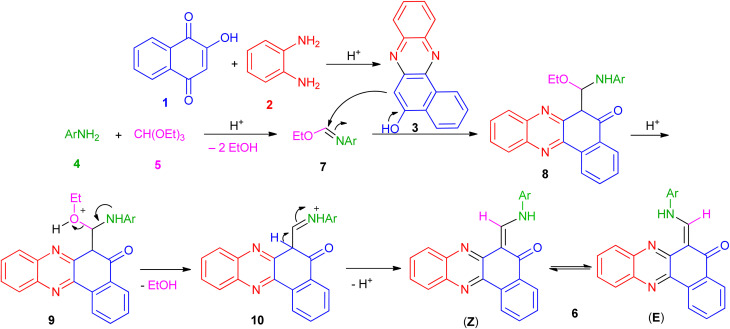
Plausible mechanism for 6-((arylamino)methylene)benzo[*a*]phenazin-5(6*H*)-one derivatives 6a–i.

Based on the above experimental results, the proposed mechanism is depicted in Scheme 1. Initially, the condensation of 2-hydroxynaphthalene-1,4-dione (1) and diamine (2) in the presence of the acid takes place to afford the intermediate 3. Also, the condensation of aromatic amine (4) with triethyl orthoformate (5) in the presence of the acid generates the corresponding intermediate 7 by loss of two ethanol molecules. After that, nucleophilic addition of 3 to the intermediate 7 gives intermediate 8 which undergo elimination of another EtOH molecule to generate intermediate 10. Finally, intermediate 10 undergo deprotonation to afford the desired product 6.

### Antimicrobial screening

#### Antibacterial activity

Newly synthesized compounds 6 were screened *in vitro* for their antibacterial activities against six bacterial strain three Gram-positive bacteria, *Staphylococcus aureus* (ATCC 29737), *Streptococcus pyogenes* (ATCC 8668), and *Bacillus subtilis* (ATCC 6633), and three Gram-negative bacteria, *Escherichia coli* (ATCC 10536), *Pseudomonas aeruginosa* (ATCC 27853), *and Klebsiella pneumoniae* (ATCC 10031) using 200 μg mL^−1^ of the compounds through the agar well diffusion method.^28^ Antimicrobial activity was measured based on the diameter of inhibition zone in mm. Gentamicin and Imipenem (10 μg per disk) were used as a standard antibiotic for the evaluation of the antimicrobial activity. Compounds 6a, 6c and 6i did not show any antimicrobial activity against any of the tested bacteria, while compounds 6b, 6d, 6e, 6f, 6g and 6h exhibited antibacterial activity. Compound 6e showed antibacterial effect against some bacteria (*S. aureus*, *B. subtilis* and *K. pneumoniae*) while it was ineffective against the others. The effective compounds showed the weak activity against Gram-positive and Gram-negative bacteria at 200 μg mL^−1^ concentration as compared to the standard drugs, *Imipenem* and Gentamicin. Compound 6b showed the maximum antibacterial activity with the inhibition zone diameter of 16 mm against *B. subtilis* (Table 4).

**Table 4:** Antibacterial activity of compounds 6^a^

Compound	*S. aureus*	*S. pyogenes*	*B. subtilis*	*E. coli*	*P. aeruginosa*	*K. pneumoniae*
6a	ND	ND	ND	ND	ND	ND
6b	7	5	16	9	6	12
6c	ND	ND	ND	ND	ND	ND
6d	11	7	6	6	9	12
6e	10	ND	6	ND	ND	9
6f	7	8	6	5	8	10
6g	6	8	7	9	8	9
6h	5	7	8	5	5	9
6i	ND	ND	ND	ND	ND	ND
Imipenem	33	32	23	27	34	38
Gentamicin	27	39	30	23	30	17

a ND: not detected. Solvent: DMSO.

#### Antifungal activity

Newly synthesized compounds 6 were screened for their antifungal against *Candida albicans* (ATCC 10231), *Aspergillus niger* (ATCC 12846) and *Rhizopus oryzae* (ATCC 9363). *Clotrimazole* and Nystatin (10 μg per disk) were used as a reference to evaluate the potency of the tested chemicals. Amongst the synthesized compounds screened for the antifungal activity, compounds 6b and 6d were toxic against to the three fungi at 200 μg mL^−1^ concentrations. Compound 6d was more effective than 6b against the fungi. The other compounds showed no inhibition zones against the tested organisms (Table 5).

**Table 5:** Antifungal activity of compounds 6

Compound	*C. albicans*	*A. niger*	*R. oryzae*
6b	10	10	7
6d	15	13	16
Clotrimazole	28	32	31
Nystatin	33	30	27

#### Antibiofilm activity

Bacterial biofilms are clusters of bacteria that are attached to each other and a surface and embedded in a self-produced polymer matrix mainly composed of polysaccharides (alginate), secreted proteins (fibrin), and extra cellular DNAs. The biofilm prevents the penetration of antimicrobial agents, and subsequently causes the bacterial resistance against the antibacterial agents such as antibiotics.^29^ The effects of the compounds on the biofilm formation inhibition of six pathogenic bacteria were determined by microtiter plate method. The biofilm formation was analyzed after incubation of the bacterial strains using 200 μg mL^−1^ of the compounds for 24 hours. The biofilm formation inhibition was determined through reading the well absorption at 630 nm.^30^ The efficiency of the effective compounds against bacteria was investigate to inhibit biofilm formation. The efficiency (%) of the effective compounds 6 on bacterial strains to inhibit biofilm formation was shown in Table 6. As shown in Table 6, the selected compounds (6b, 6d, 6e, 6f, 6g and 6h) showed the moderate anti biofilm activity against six bacterial strains. Compound 6b had the most efficiency to inhibit biofilm formation of *Bacillus subtilis* (80%) at 200 μg mL^−1^ concentration.

**Table 6:** Antibiofilm activity of compounds 6^a^

Compound	*S. aureus*	*S. pyogenes*	*B. subtilis*	*E. coli*	*P. aeruginosa*	*K. pneumoniae*
6b	32	48	80	32	38	55
6d	76	66	22	50	52	60
6e	70	10	17	ND	ND	35
6f	34	55	41	44	29	40
6g	11	50	63	44	27	42
6h	23	49	54	19	15	37

a ND: not detected.

## Conclusions

In summary, we have described an efficient and environmentally friendly protocol for the synthesis of novel 6-((arylamino)methylene)benzo[*a*]phenazin-5(6*H*)-one derivatives from a one-pot, four-component condensation of 2-hydroxy-1,4-naphthoquinone, 1,2-phenylenediamine, triethyl orthoformate and different aromatic amines using formic acid as catalyst under solvent-free conditions. The advantages of this protocol are operational simplicity, metal-free, good to high yields, chromatography-free purification, broad substrate scope and ready availability of the starting materials. The ^1^H-NMR spectra of the synthesized compounds revealed that they exist keto-enamine form and undergo *Z*/*E*-isomerization around the C=C bond in DMSO-*d*_6_ at room temperature. Antibacterial activities of the synthesized compounds against *S. aureus*, S. *pyogenes*, *B. subtilis*, *E coli*, *P. aeruginosa*, *K. pneumoniae* bacteria were assayed. Some of the compounds inhibited the growth of tested bacteria at the concentration of 200 μg mL^−1^. Compound 6b showed the maximum antibacterial activity. Antifungal activity of thesynthesized compounds against *C. albicans*, *A. niger* and *R. oryzae fungi* were tested. Compound 6d exhibited the maximum antifungal activity compared to the reference antimicrobial agents. It should be noted that these compounds showed the weak or no antibacterial and antifungal activity at 200 μg mL^−1^ concentration, while they might be more effective against bacteria and fungi at higher concentrations. In addition, we have explored antibiofilm activity against six bacterial strains of the target compounds. The obtained results have shown that compound 6b has the most efficiency to inhibit biofilm formation of *B. subtilis* (80%) at 200 μg mL^−1^ concentration.

## Experimental

### General information

All commercially available chemicals and reagents were used without further purification. Melting points were determined with an Electrothermal model 9100 apparatus and are uncorrected. FT-IR spectra were recorded on a Shimadzu 4300 spectrophotometer. The ^1^H-NMR spectra were recorded in DMSO-d6 on Bruker DRX-300 Avance spectrometers. Chemical shifts (*δ*) are reported in parts per million and are referenced to the NMR solvent. Mass spectra of the products were obtained with a HP (Agilent technologies) 5973 Mass Selective Detector.

#### General procedure for the synthesis of compounds 6a–i

A mixture of 2-hydroxynaphthalene-1,4-dione (1, 1.0 mmol), benzene-1,2-diamine (2, 1.0 mmol), and formic acid (10 mo%) was stirred for 15 min at 90 °C until an orange solid of benzo[*a*]phenazin-5-ol (3) was formed. After completion of the first step of reaction (monitored by TLC), amine (4, 1.0 mmol), triethyl orthoformate (5, 1.0 mmol) were added into the reaction mixture and stirred at 90 °C under solvent-free conditions for an appropriate time, and the progress of the reaction was monitored by TLC (*n*-hexane/ethyl acetate: 1 : 1). After completion of the reaction as indicated in Table 2, the mixture was cooled down to room temperature and CH_3_CN (3 mL) was added. Then, the mixture was stirred for 3 min at 80 °C and after cooling, the resulting precipitate was filtered and the pure product was obtained.

#### 6-(((6-Methylpyridin-2-yl)amino)methylene)benzo[*a*]phenazin-5(6*H*)-one (6a)

Yellow powder; M.P. = 196–197 °C; IR (KBr, cm^−1^): 3396, 3068, 2924, 1656, 1605, 1552, 1527, 1455, 1428, 1398, 1326, 1281, 1167; ^1^H-NMR (300 MHz, DMSO-*d*_6_): *δ* = 2.55 (s, 3H, CH_3_), 7.09–7.17 (m, 1H, Ar–H), 7.33–7.36 (m, 1H, Ar–H), 7.68–7.86 (m, 5H, Ar–H), 7.99–8.08 (m, 2H, Ar–H), 8.21 (d, 1H, *J* = 7.8 Hz, Ar–H), 8.78 (d, 0.6H, *J* = 7.8 Hz, Ar–H), 9.90 (d, 0.4H, *J* = 7.8 Hz, Ar–H), 9.47 (d, 0.6H, *J* = 12.9 Hz, CH), 10.04 (d, 0.4H, *J* = 12.9 Hz, CH), 13.67 (d, 0.4H, *J* = 12.9 Hz, NH), 13.73 (d, 0.6H, *J* = 12.9 Hz, NH); ^1^H-NMR (300 MHz, DMSO-*d*_6_ + D_2_O): *δ* = 2.53 (s, 3H, CH_3_), 7.10–7.32 (m, 2H, Ar–H), 7.25–7.32 (m, 1H, Ar–H), 7.69–7.88 (m, 5H, Ar–H), 8.00–8.10 (m, 2H, Ar–H), 8.21 (t, 1H, *J* = 8.1 Hz, Ar–H), 8.79 (d, 0.6H, *J* = 8.1 Hz, Ar–H), 9.90 (d, 0.4H, *J* = 8.1 Hz, Ar–H), 9.45 (s, 0.6H, CH), 10.02 (s, 0.4H, CH); MS *m*/*z* (%): 365 (M + 1)^+^, 364 (M^+^), 335, 273, 272 (100), 256, 243, 218, 217, 216, 215, 190, 182, 164, 140, 114, 102, 94, 93, 92, 77, 76, 65.

#### 6-(((3-Methylpyridin-2-yl)amino)methylene)benzo[*a*]phenazin-5(6*H*)-one (6b)

Brown powder; M.P. = 204–206 °C; IR (KBr, cm^−1^): 3397, 3060, 2938, 1654, 1601, 1583, 1559, 1527, 1462, 1417, 1386, 1332, 1273, 1148; ^1^H-NMR (300 MHz, DMSO-*d*_6_): *δ* = 2.26 (s, 0.9H, CH_3_), 2.34 (s, 2.1H, CH_3_), 7.01–7.12 (m, 1H, Ar–H), 7.40–7.66 (m, 4H, Ar–H), 7.71–8.23 (m, 5H, Ar–H), 8.48 (d, 0.7H, *J* = 8.1 Hz, Ar–H), 8.66 (d, 0.3H, *J* = 8.1 Hz, Ar–H), 9.21 (d, 0.7H, *J* = 12.3 Hz, CH), 9.83 (d, 0.3H, *J* = 12.0 Hz, CH), 13.36 (d, 0.7H, *J* = 12.3 Hz, NH), 13.90 (d, 0.3H, *J* = 12.0 Hz, NH); MS *m*/*z* (%): 365 (M + 1)^+^, 364 (M^+^), 349, 347, 336, 335, 292, 273, 272 (100), 243, 218, 217, 216, 215, 190, 182, 167, 140, 119, 102, 93, 92, 77, 76, 65.

#### 6-(((4-Methylpyridin-2-yl)amino)methylene)benzo[*a*]phenazin-5(6*H*)-one (6c)

Brown powder; M.P. = 173–174 °C; IR (KBr, cm^−1^): 3396, 3060, 2920, 1651, 1628, 1599, 1544, 1532, 1484, 1399, 1344, 1281, 1143; ^1^H-NMR (300 MHz, DMSO-*d*_6_): *δ* = 2.28 (s, 1.2H, CH_3_), 2.31 (s, 1.8H, CH_3_), 7.01–7.11 (m, 1H, Ar–H), 7.33 (s, 0.4H, Ar–H), 7.40 (s, 0.6H, Ar–H), 7.69–7.71 (m, 4H, Ar–H), 7.96–8.30 (m, 4H, Ar–H), 8.76 (d, 0.6H, *J* = 7.8 Hz, Ar–H), 8.86 (d, 0.4H, *J* = 8.1 Hz, Ar–H), 9.47 (d, 0.6H, *J* = 12.6 Hz, CH), 10.03 (d, 0.4H, *J* = 12.6 Hz, CH), 13.59 (d, 0.4H, *J* = 12.6 Hz, NH), 13.67 (d, 0.6H, *J* = 12.6 Hz, NH); MS *m*/*z* (%): 365 (M + 1)^+^, 364 (M^+^), 336, 335, 292, 273, 272 (100), 243, 218, 217, 216, 215, 190, 182, 168, 140, 114, 102, 93, 92, 77, 76, 65.

#### 6-((Pyridin-3-ylamino)methylene)benzo[*a*]phenazin-5(6*H*)-one (6d)

Brown powder; M.P. = 193–194 °C; IR (KBr, cm^−1^): 3396, 3058, 1652, 1595, 1562, 1530, 1462, 1414, 1331, 1285, 1218, 1176; ^1^H-NMR (300 MHz, DMSO-*d*_6_): *δ* = 7.42–7.50 (m, 1H, Ar–H), 7.64–7.84 (m, 5H, Ar–H), 7.96–8.08 (m, 2H, Ar–H), 8.16–8.42 (m, 3H, Ar–H), 8.85–8.88 (m, 1H, Ar–H), 8.96 (d, 0.65H, *J* = 13.2 Hz, CH), 9.49 (d, 0.35H, *J* = 13.2 Hz, CH), 13.75 (d, 0.35H, *J* = 13.2 Hz, NH), 13.84 (d, 0.65H, *J* = 13.2 Hz, NH); MS *m*/*z* (%): 351 (M + 1)^+^, 350 (M^+^) (100), 333, 322, 321, 273, 272, 246, 218, 217, 216, 215, 190, 175, 164, 140, 114, 102, 89, 78, 77, 76, 51.

#### 6-((Pyridin-4-ylamino)methylene)benzo[*a*]phenazin-5(6*H*)-one (6e)

Brown powder; M.P. = 249–251 °C; IR (KBr, cm^−1^): 3338, 3063, 1652, 1589, 1549, 1526, 1439, 1414, 1326, 1280, 1205, 1179; ^1^H-NMR (300 MHz, DMSO-*d*_6_): *δ* = 7.52–7.77 (m, 6H, Ar–H), 7.92–7.98 (m, 1H, Ar–H), 8.11–8.22 (m, 2H, Ar–H), 8.49–8.53 (m, 2H, Ar–H), 8.66 (d, 0.68H, *J* = 8.1 Hz, Ar–H), 8.77 (d, 0.32H, *J* = 7.8 Hz, Ar–H), 8.89 (d, 0.68H, *J* = 12.9 Hz, CH), 9.43 (d, 0.32H, *J* = 12.9 Hz, CH), 13.38 (d, 0.32H, *J* = 12.9 Hz, NH), 13.60 (d, 0.68H, *J* = 12.9 Hz, NH); MS *m*/*z* (%): 351 (M + 1)^+^, 350 (M^+^), 333, 322, 321, 273, 272 (100), 246, 243, 218, 217, 216, 215, 190, 175, 164, 140, 212, 114, 102, 89, 78, 77, 51.

#### 6-((Pyrimidin-2-ylamino)methylene)benzo[*a*]phenazin-5(6*H*)-one (6f)

Yellow powder; M.P. = 225–226 °C; IR (KBr, cm^−1^): 3396, 3061, 1690, 1662, 1591, 1551, 1442, 1407, 1363, 1293, 1249, 1176, 1138; ^1^H-NMR (300 MHz, DMSO-*d*_6_): *δ* = 7.36–7.39 (m, 1H, Ar–H), 7.75–7.81 (m, 3H, Ar–H), 7.95–8.09 (m, 4H, Ar–H), 8.74–8.77 (m, 3H, Ar–H), 9.09 (d, 0.42H, *J* = 13.2 Hz, CH), 9.26 (d, 0.58H, *J* = 13.2 Hz, CH), 12.43 (d, 0.42H, *J* = 13.2 Hz, NH), 12.52 (d, 0.58H, *J* = 13.2 Hz, NH); MS *m*/*z* (%): 352 (M + 1)^+^, 351 (M^+^), 322, 280, 279, 273, 272, 251, 223, 222 (100), 206, 195, 194, 190, 175, 172, 168, 140, 129, 116, 115, 114, 104, 102, 101, 97, 89, 80, 79, 77, 76, 75, 53.

#### 6-(((4-Chlorophenyl)amino)methylene)benzo[*a*]phenazin-5(6*H*)-one (6g)

Brown powder; M.P. = 218–220 °C; IR (KBr, cm^−1^): 3401, 3059, 1661, 1596, 1566, 1529, 1461, 1438, 1412, 1338, 1290, 1213, 1092; ^1^H-NMR (300 MHz, DMSO-*d*_6_): *δ* = 7.33–7.47 (m, 2H, Ar–H), 7.56–7.83 (m, 5H, Ar–H), 7.90–8.03 (m, 2H, Ar–H), 8.13–8.25 (m, 2H, Ar–H), 8.70–8.88 (m, 1H, Ar–H), 9.19 (d, 0.70H, *J* = 12.9 Hz, CH), 9.40 (d, 0.30H, *J* = 13.2 Hz, CH), 12.85 (d, 0.30H, *J* = 13.2 Hz, NH), 13.79 (d, 0.70H, *J* = 12.9 Hz, NH); MS *m*/*z* (%): 385 (M + 2)^+^, 384 (M + 1)^+^, 383 (M^+^), 313, 312, 311, 285, 284, 283, 282, 273, 272, 254, 249, 248 (100), 246, 238, 218, 217, 190, 165, 157, 151, 129, 127, 113, 111, 105, 104, 101, 89, 77, 76, 75, 63, 51.

#### 6-((*p*-Tolylamino)methylene)benzo[*a*]phenazin-5(6*H*)-one (6h)

Orange powder; M.P. = 194–196 °C; IR (KBr, cm^−1^): 3397, 3055, 2915, 1653, 1595, 1563, 1529, 1437, 1400, 1340, 1280, 1235, 1172, 1114; ^1^H-NMR (300 MHz, DMSO-*d*_6_): *δ* = 2.24 (s, 3H, CH_3_), 7.19–7.24 (m, 2H, Ar–H), 7.42–7.47 (m, 2H, Ar–H), 7.63–7.81 (m, 4H, Ar–H), 7.93–8.02 (m, 1H, Ar–H), 8.14–8.29 (m, 2H, Ar–H), 8.75 (d, 0.60H, *J* = 7.8 Hz, Ar–H), 8.86 (d, 0.40H, *J* = 7.8 Hz, Ar–H), 8.92 (d, 0.40H, *J* = 13.5 Hz, CH), 9.46 (d, 0.60H, *J* = 13.2 Hz, CH), 12.86 (d, 0.40H, *J* = 13.5 Hz, NH), 13.93 (d, 0.60H, *J* = 13.2 Hz, NH); MS *m*/*z* (%): 364 (M + 1)^+^, 363 (M^+^) (100), 346, 334, 319, 273, 272, 246, 218, 217, 190, 181, 164, 140, 118, 91, 89, 77, 76, 65.

#### 6-(((4-Methoxyphenyl)amino)methylene)benzo[*a*]phenazin-5(6*H*)-one (6i)

Orange powder; M.P. = 239–240 °C; IR (KBr, cm^−1^): 3432, 3055, 2930, 1650, 1600, 1563, 1514, 1461, 1438, 1400, 1344, 1284, 1254, 1171; ^1^H-NMR (300 MHz, DMSO-*d*_6_): *δ* = 3.68 (s, 1.33H, OCH_3_), 3.71 (s, 1.67H, OCH_3_), 6.94–6.99 (m, 2H, Ar–H), 7.47 (d, 2H, *J* = 8.7 Hz, Ar–H), 7.57–7.76 (m, 3H, Ar–H), 7.89–7.99 (m, 2H, Ar–H), 8.08–8.26 (m, 2H, Ar–H), 8.69–8.84 (m, 1.60H, Ar–H, CH), 9.34 (d, 0.40H, *J* = 13.2 Hz, CH), 13.84 (d, 0.40H, *J* = 13.2 Hz, NH), 13.98 (d, 0.60H, *J* = 13.2 Hz, NH); MS *m*/*z* (%): 380 (M + 1)^+^, 379 (M^+^) (100), 365, 364, 362, 335, 308, 273, 272, 246, 243, 242, 218, 217, 189, 164, 140, 122, 102, 92, 77, 64.

## Conflicts of interest

There are no conflicts of interest to declare.

## Supplementary Material

RA-013-D3RA05788G-s001
