# Supplementary material for: Design, synthesis, antimicrobial, antibiofilm evaluation and Z/E-isomerization of novel 6-((arylamino)methylene)benzo[a] phenazin-5(6H)-ones induced by organic solvent
Source: RSC Adv. 2023 Oct 9;13(42):29393–400. doi: 10.1039/d3ra05788g (PMC10560977; doi:10.1039/d3ra05788g)
Supplement: RA-013-D3RA05788G-s001 [file RA-013-D3RA05788G-s001.pdf]

Electronic Supplementary Material (ESI) for RSC Advances.
This journal is © The Royal Society of Chemistry 2023

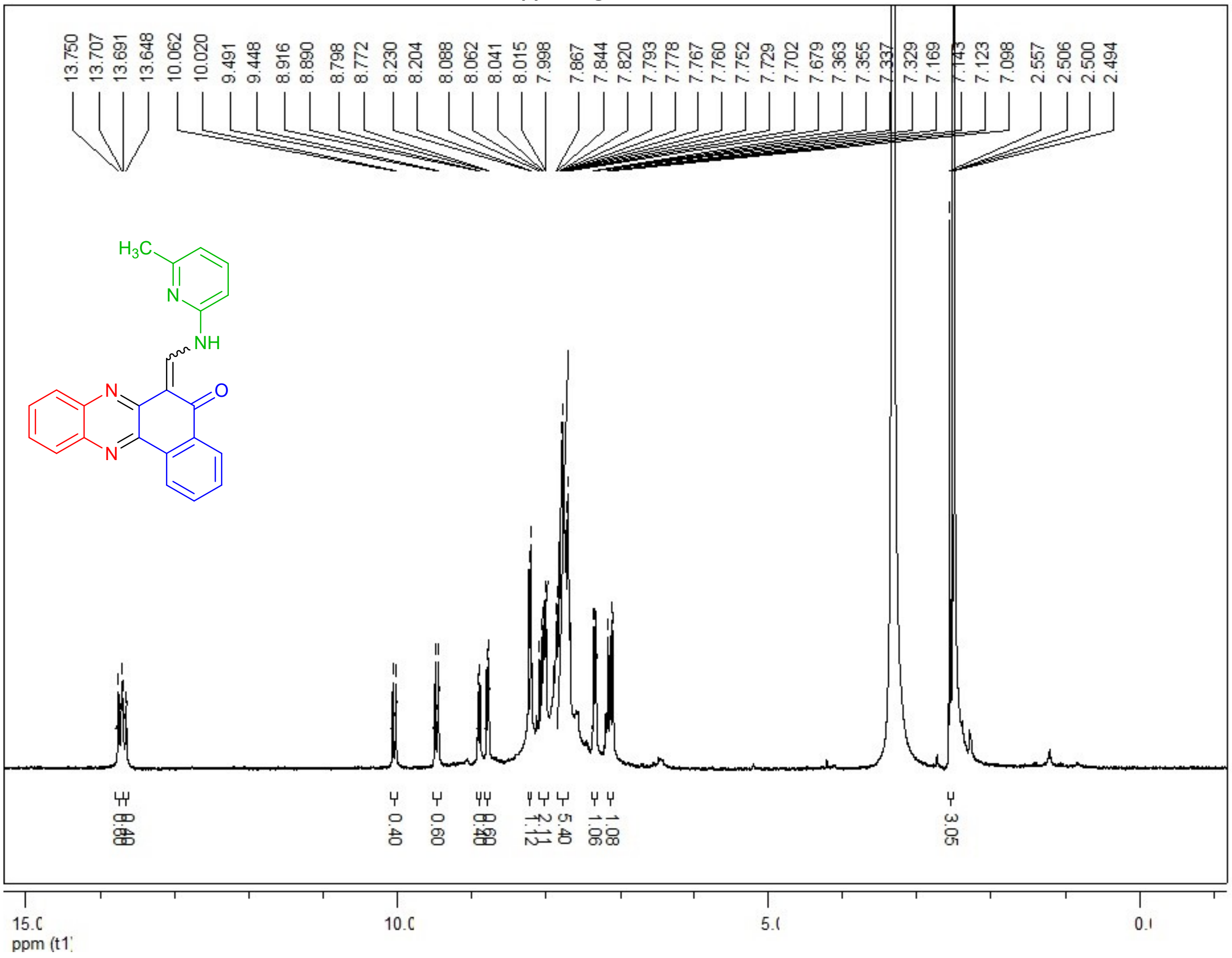

^{1}H-NMR spectrum of compound **6a** (300 MHz, DMSO-d_6)

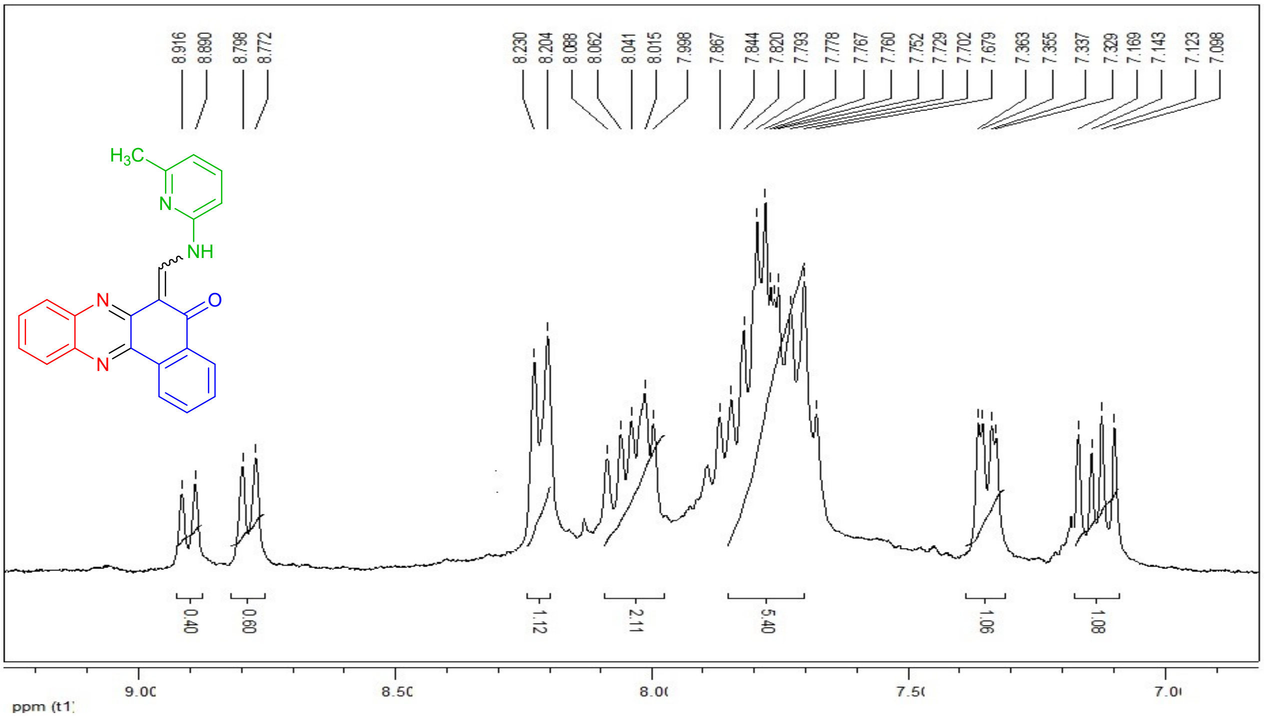

^{1}H-NMR spectrum of compound **6a** (300 MHz, DMSO-d_6)

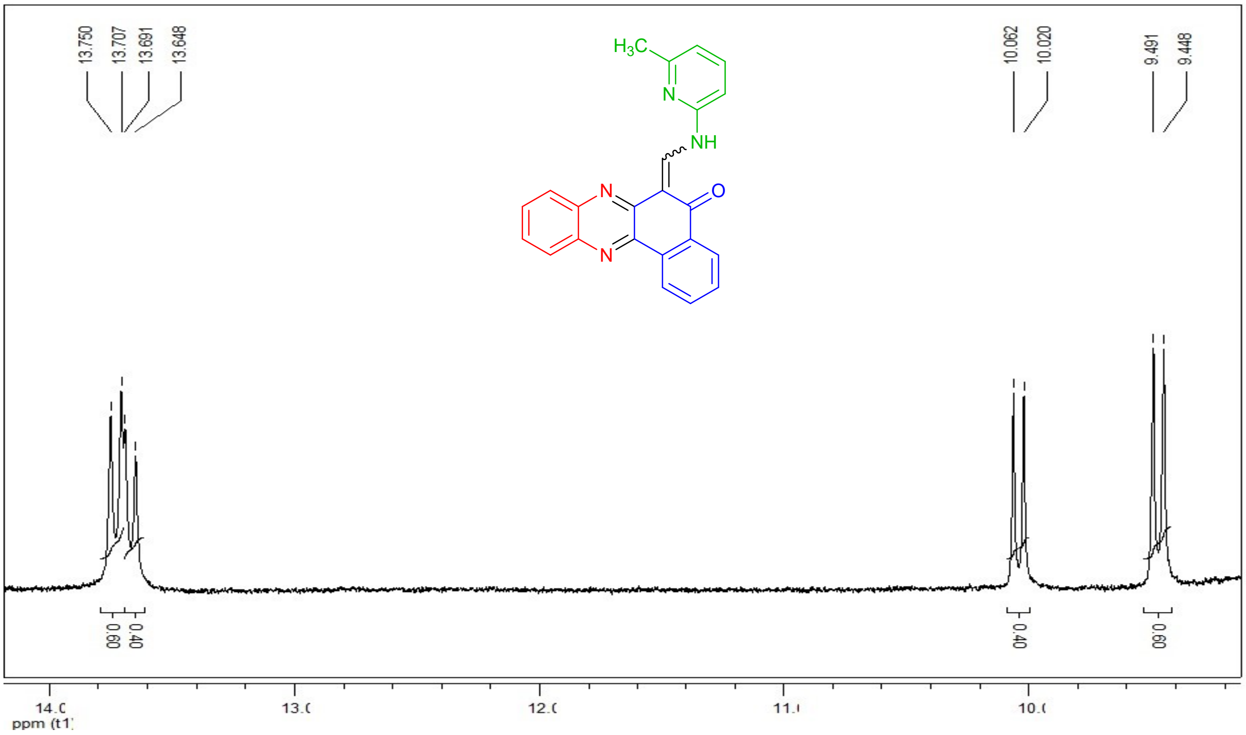

^{1}H-NMR spectrum of compound **6a** (300 MHz, DMSO-d_6)

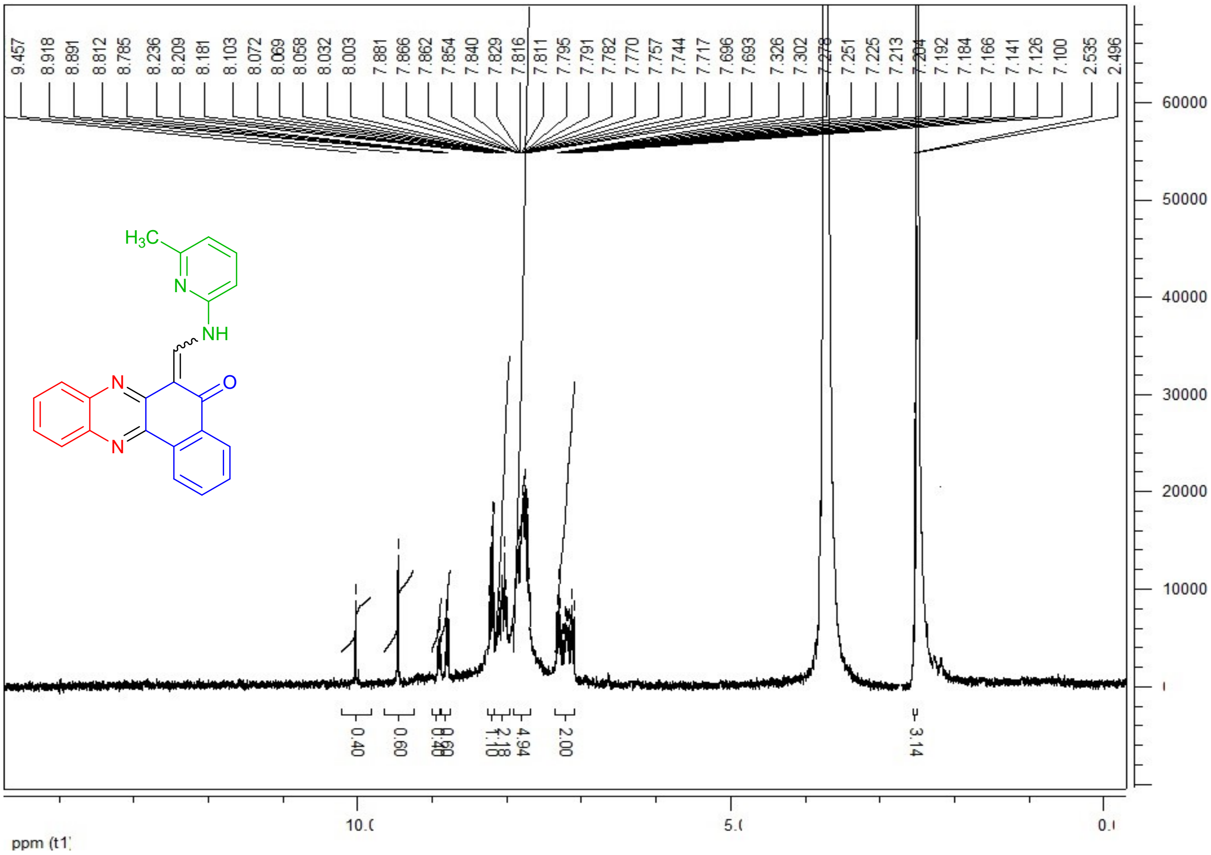

^{1}H-NMR spectrum of compound **6a** (300 MHz, DMSO-d_6 + D_2O)

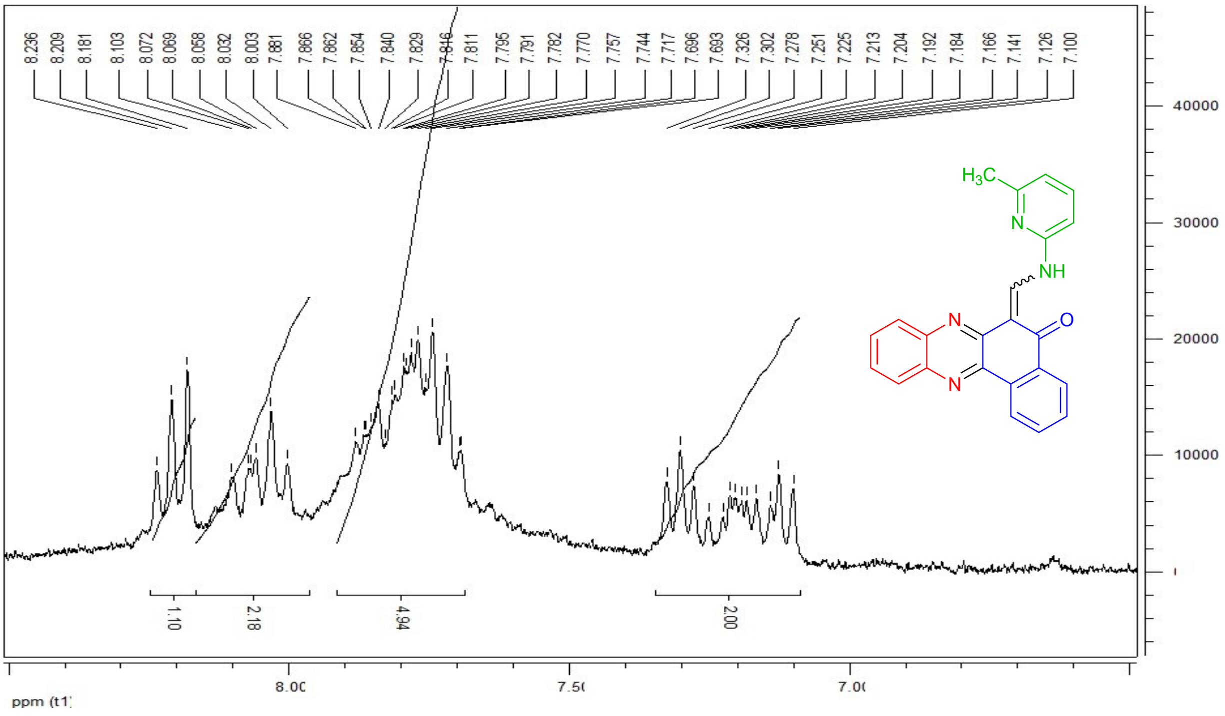

^{1}H-NMR spectrum of compound **6a** (300 MHz, DMSO-d_6 + D_2O)

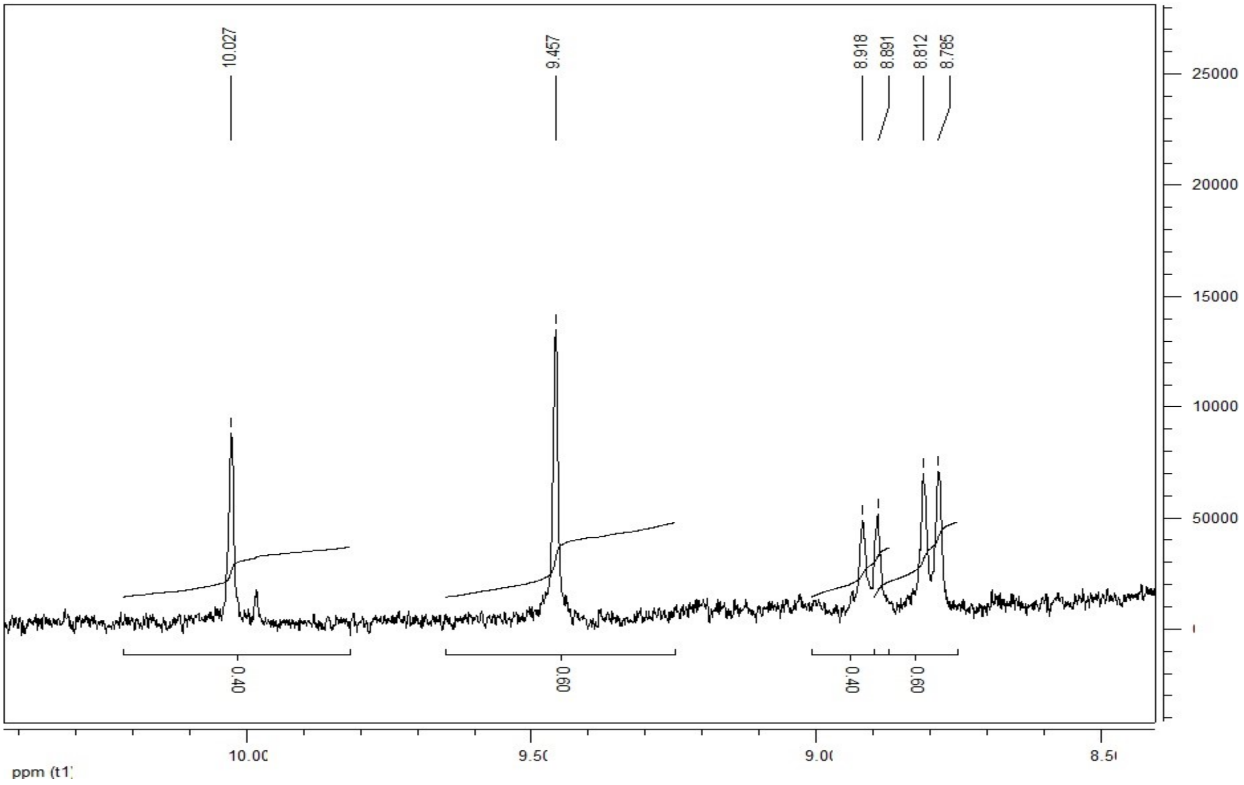

^{1}H-NMR spectrum of compound **6a** (300 MHz, DMSO-d_6 + D_2O)

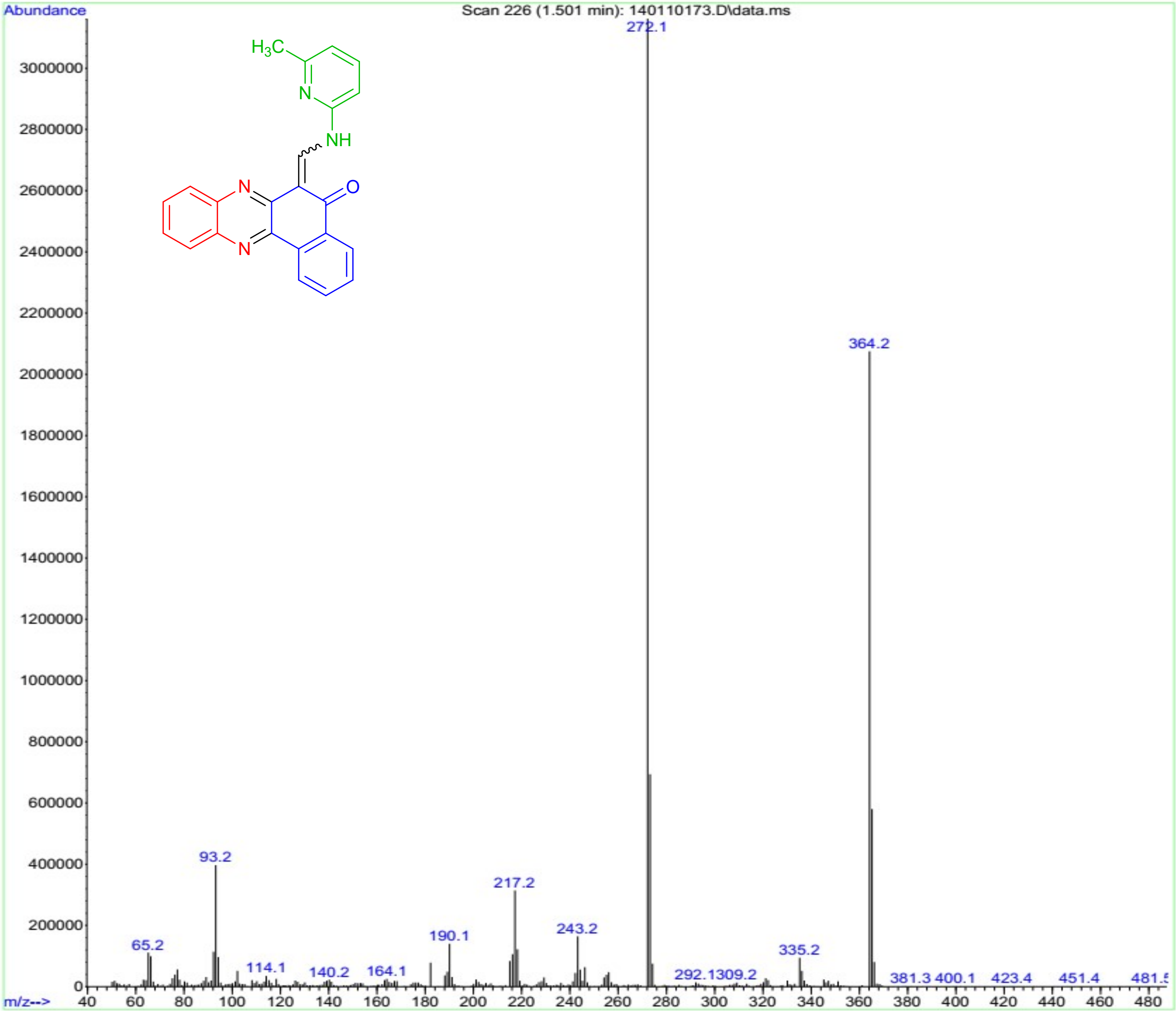

Mass spectrum of compound **6a**

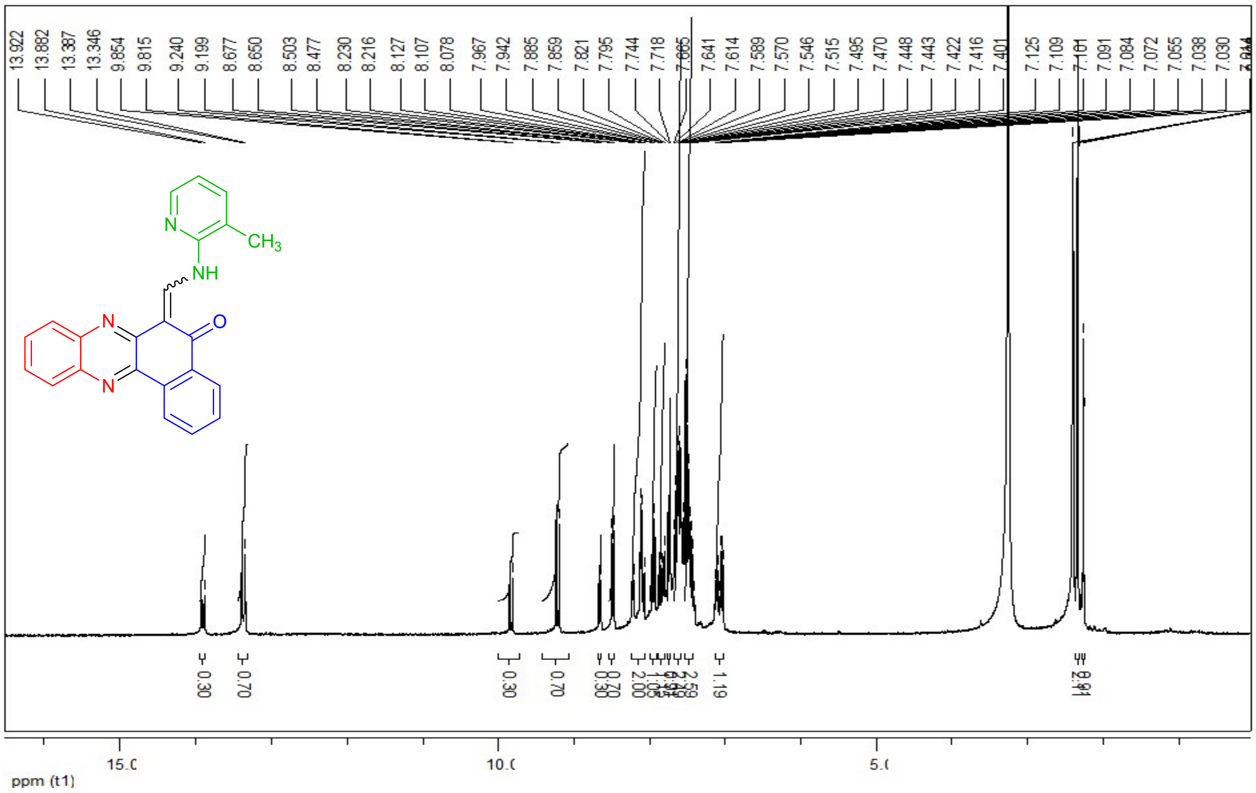

^{1}H-NMR spectrum of compound **6b** (300 MHz, DMSO-d_6)

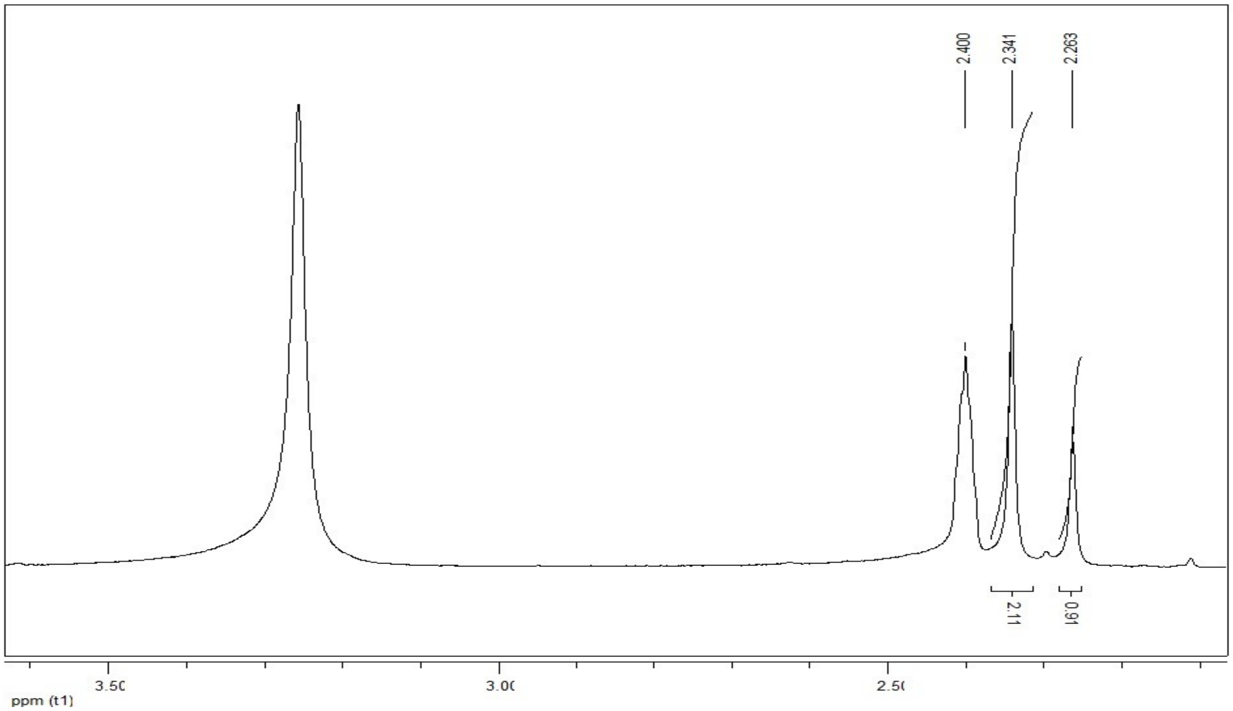

^{1}H-NMR spectrum of compound **6b** (300 MHz, DMSO-d_6)

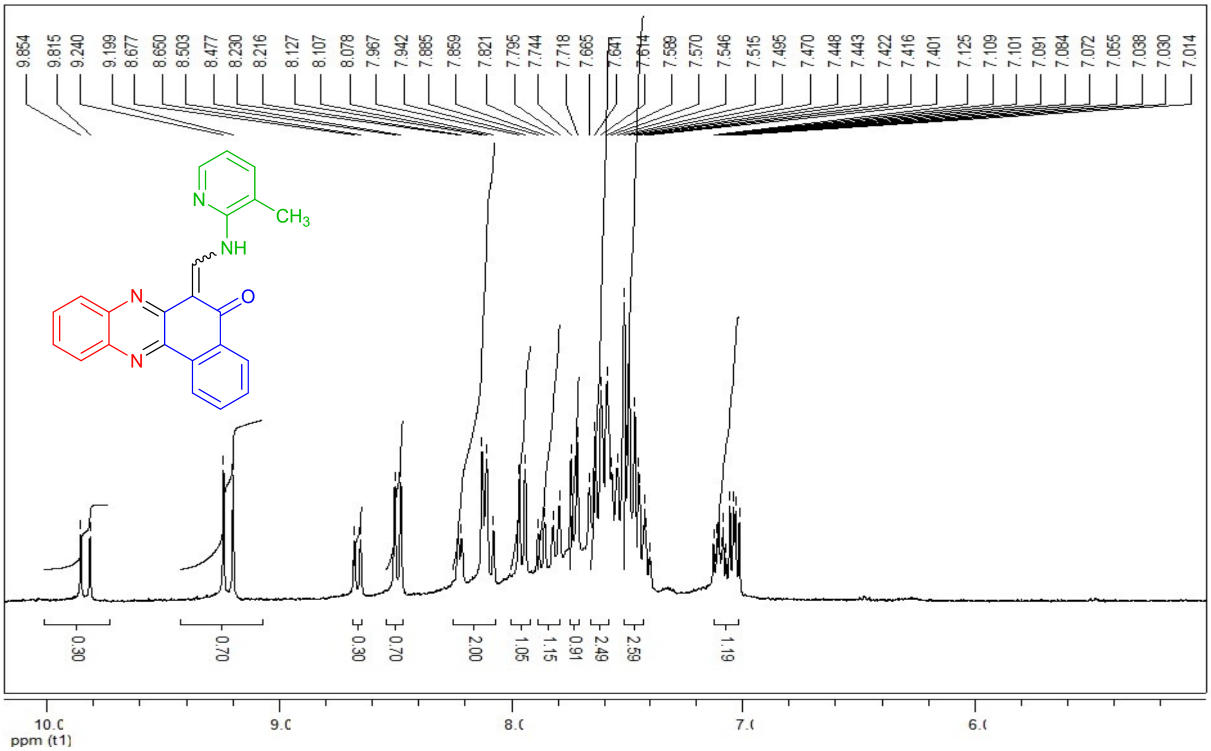

^{1}H-NMR spectrum of compound **6b** (300 MHz, DMSO-d_6)

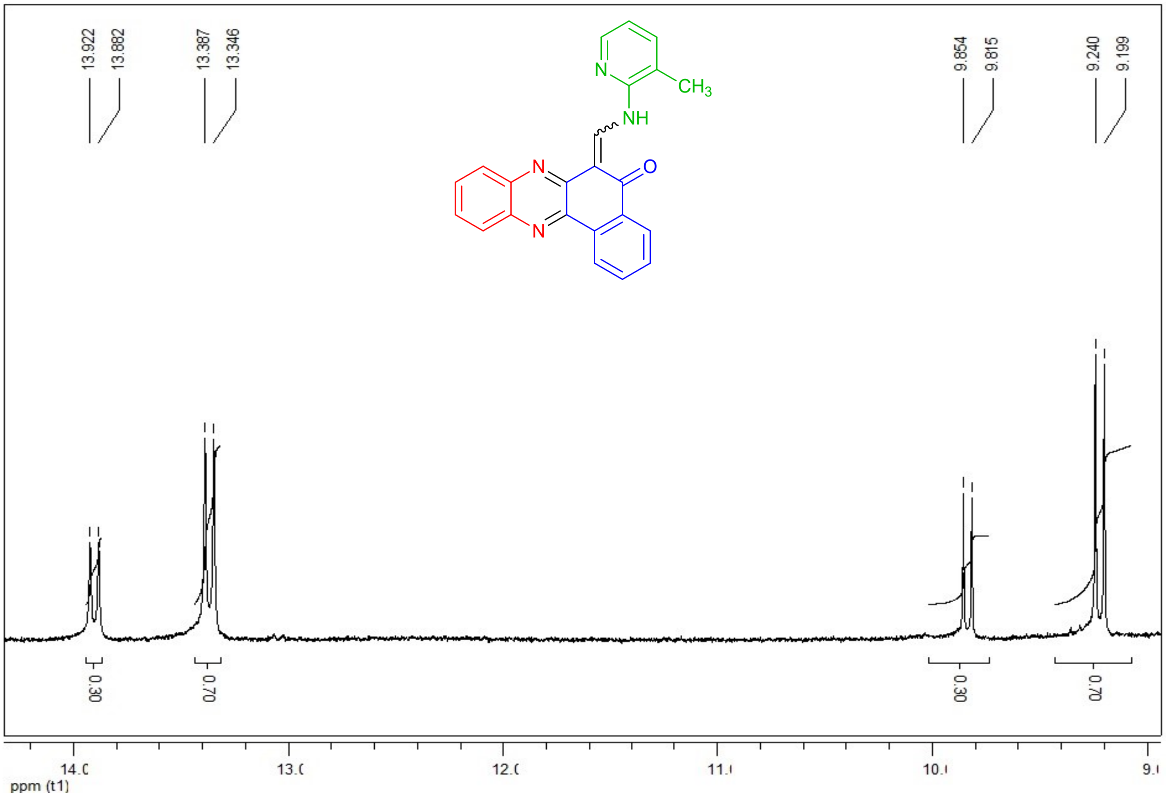

^{1}H-NMR spectrum of compound **6b** (300 MHz, DMSO-d_6)

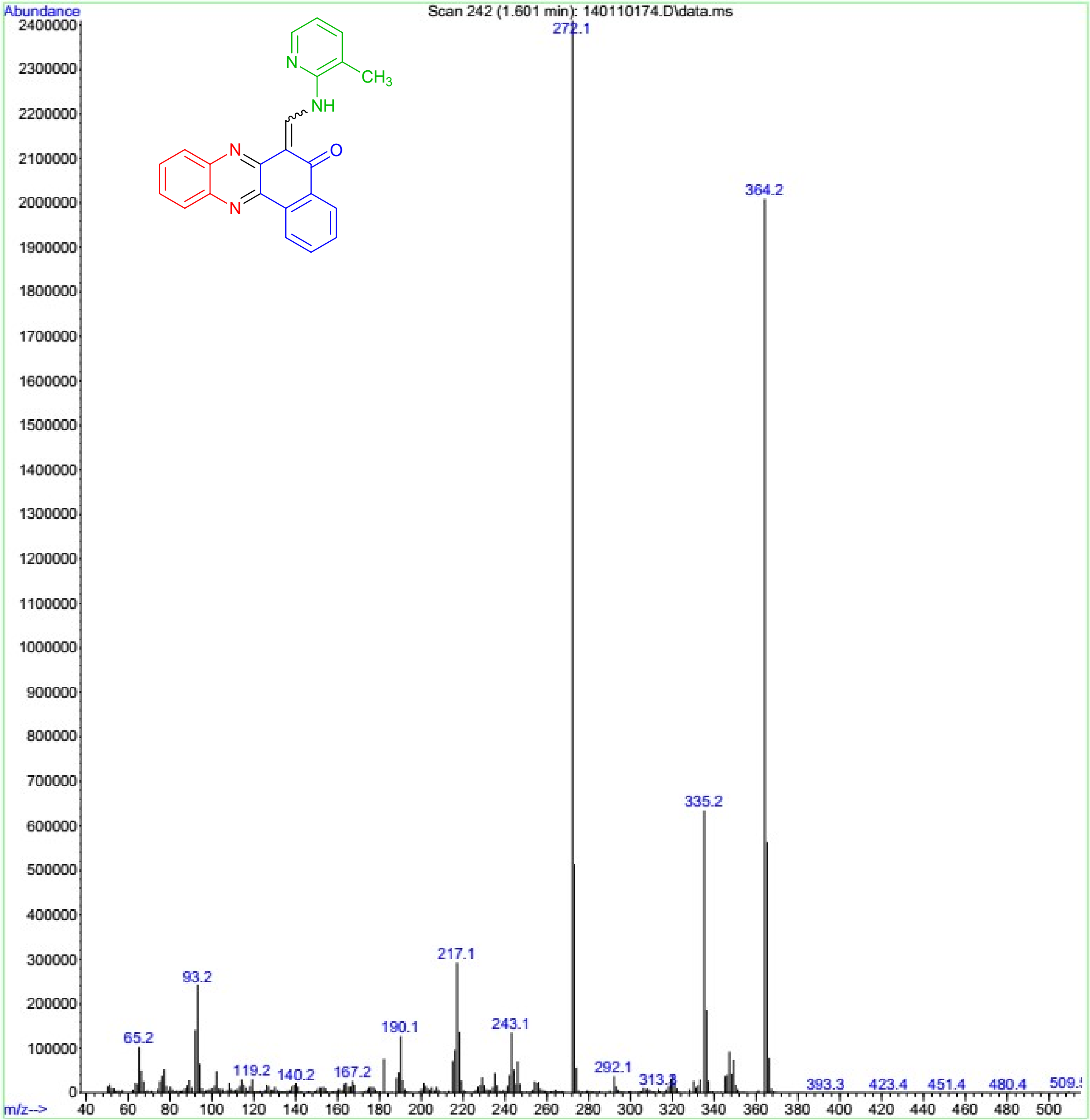

Mass spectrum of compound **6b**

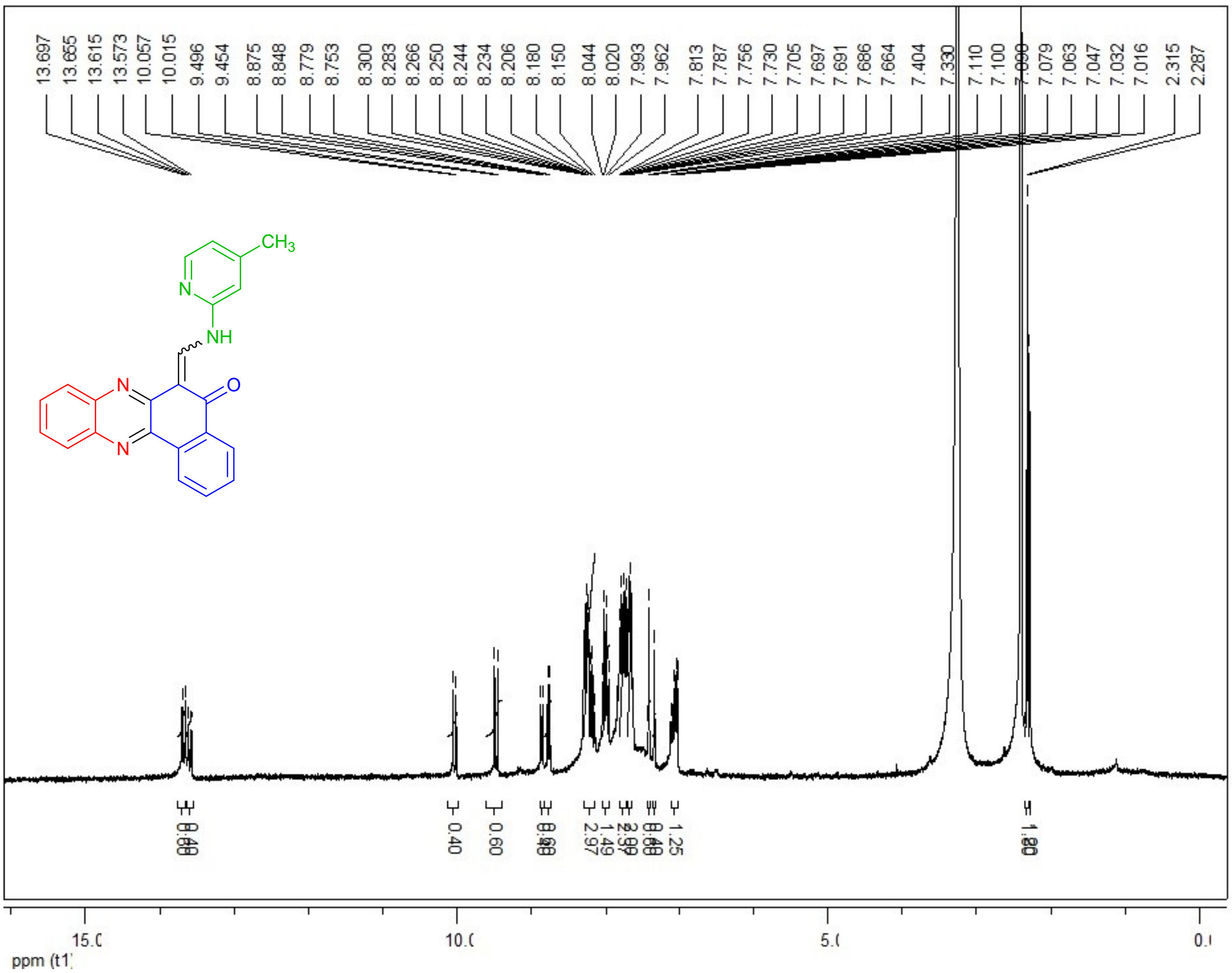

^{1}H-NMR spectrum of compound **6c** (300 MHz, DMSO-d_6)

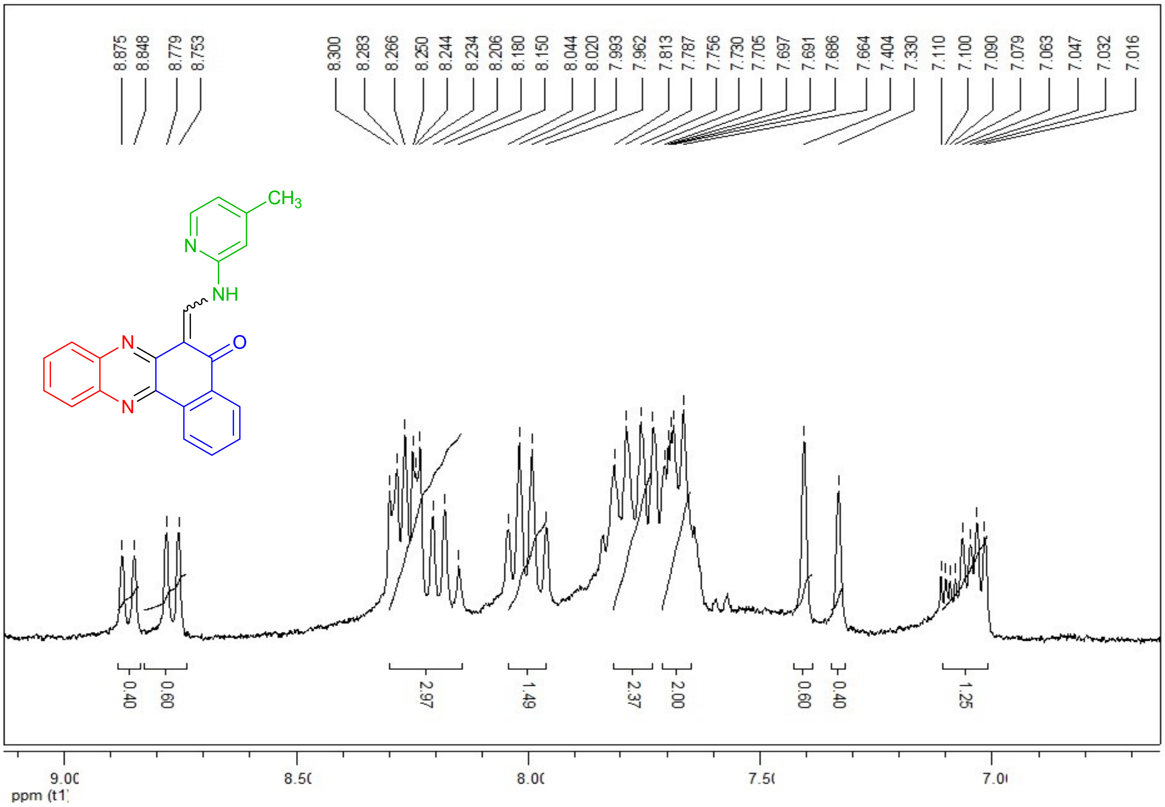

^{1}H-NMR spectrum of compound **6c** (300 MHz, DMSO-d_6)

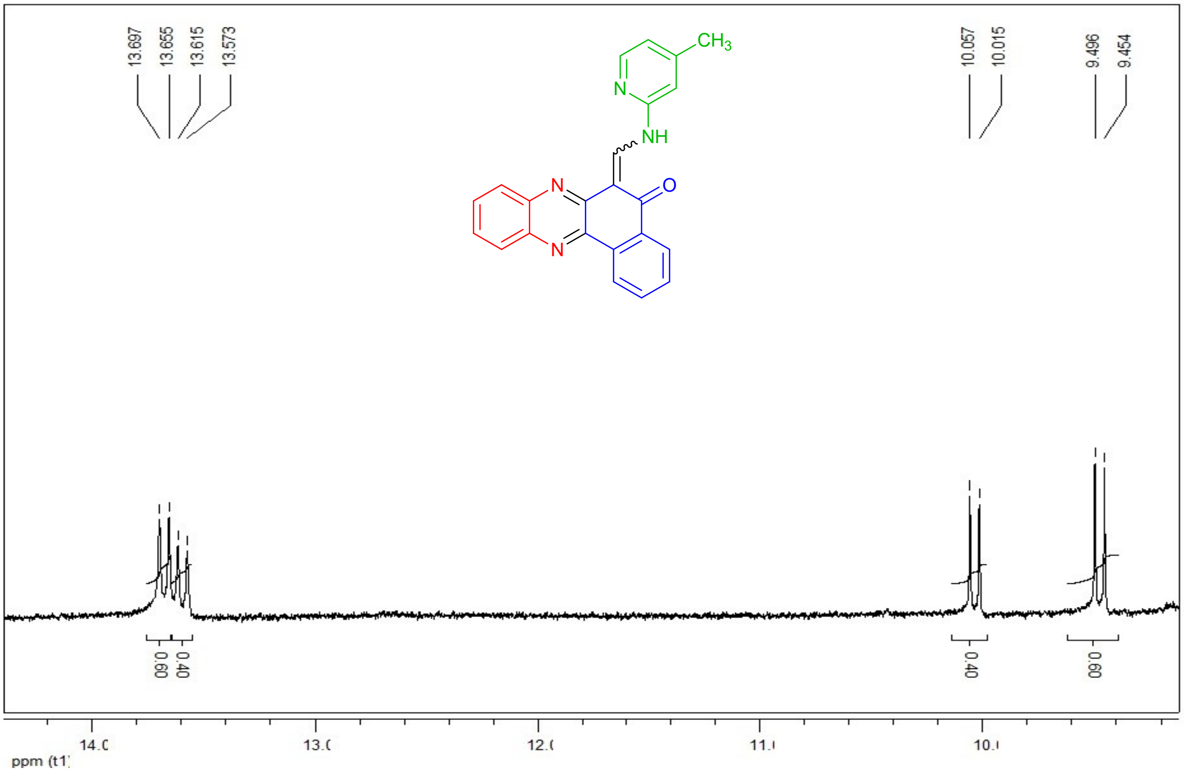

^{1}H-NMR spectrum of compound **6c** (300 MHz, DMSO-d_6)

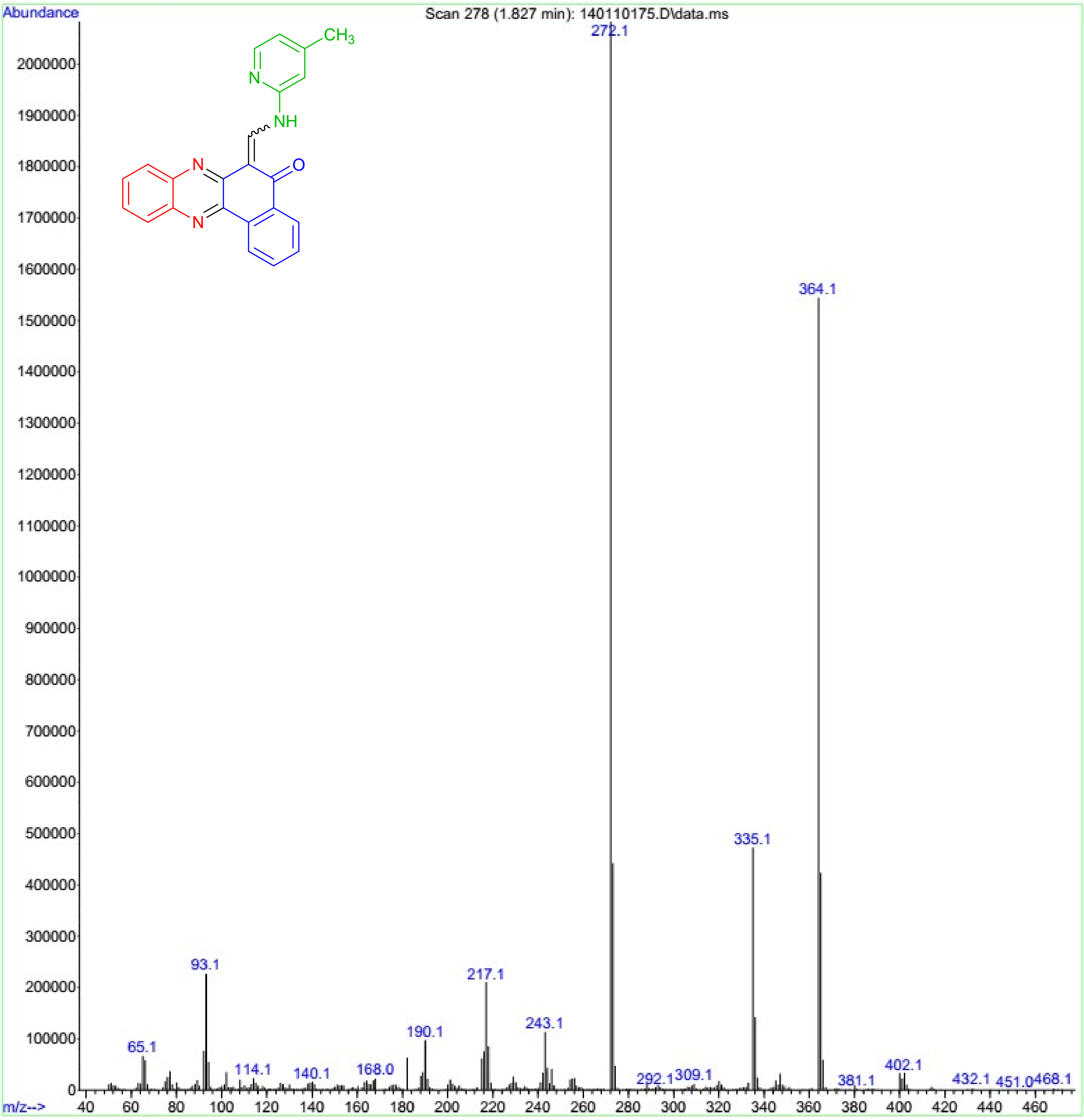

Mass spectrum of compound **6c**

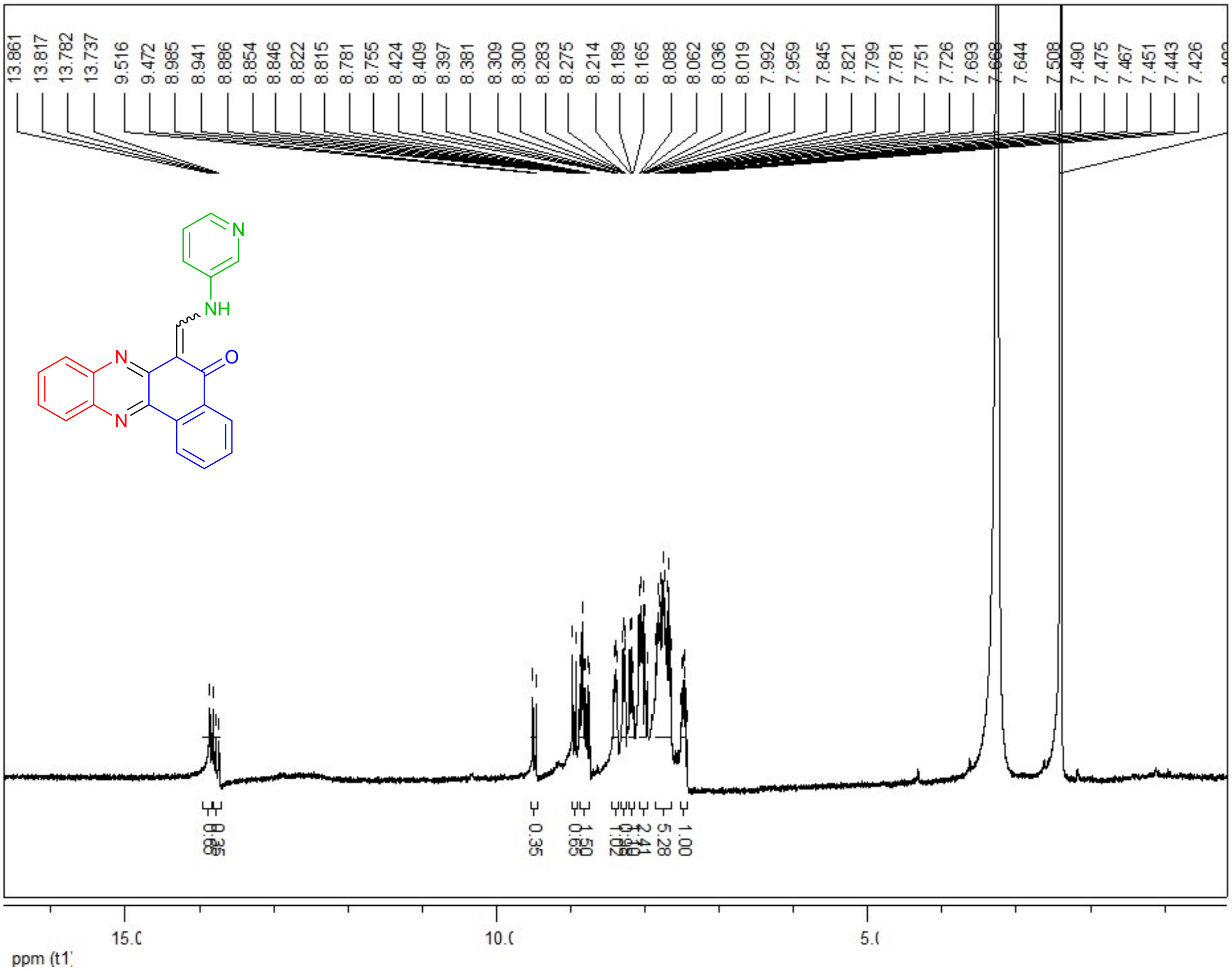

^{1}H-NMR spectrum of compound **6d** (300 MHz, DMSO-d_6)

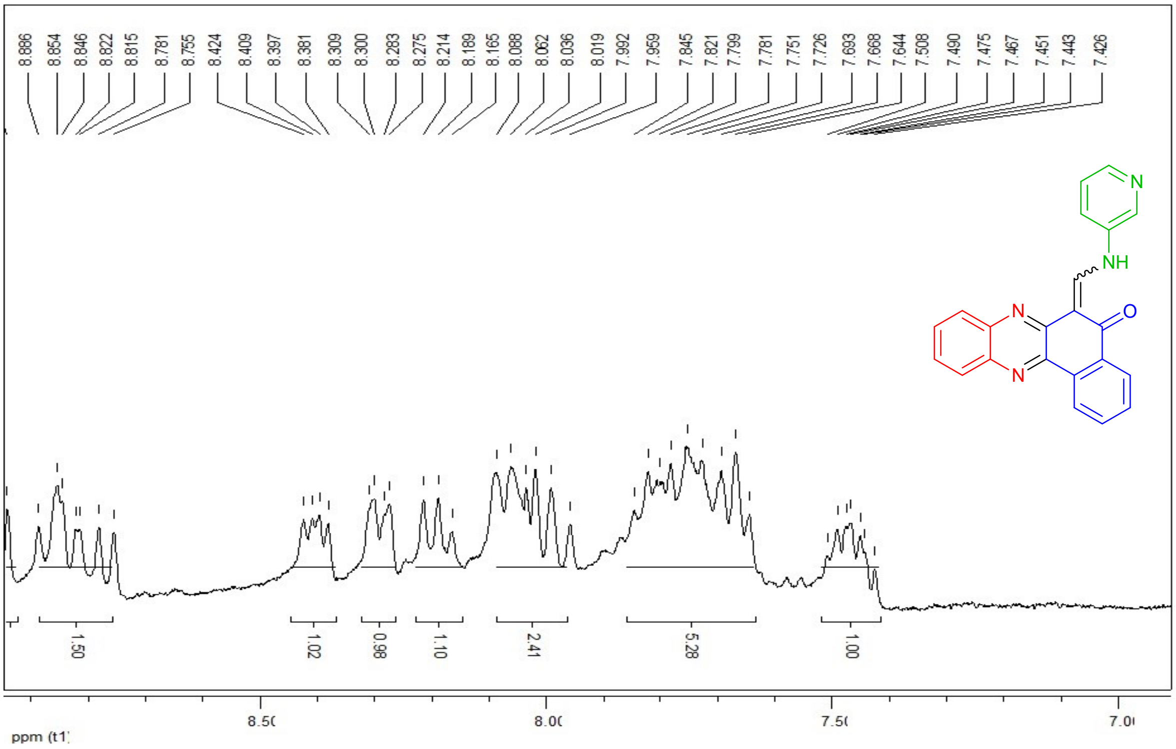

^{1}H-NMR spectrum of compound **6d** (300 MHz, DMSO-d_6)

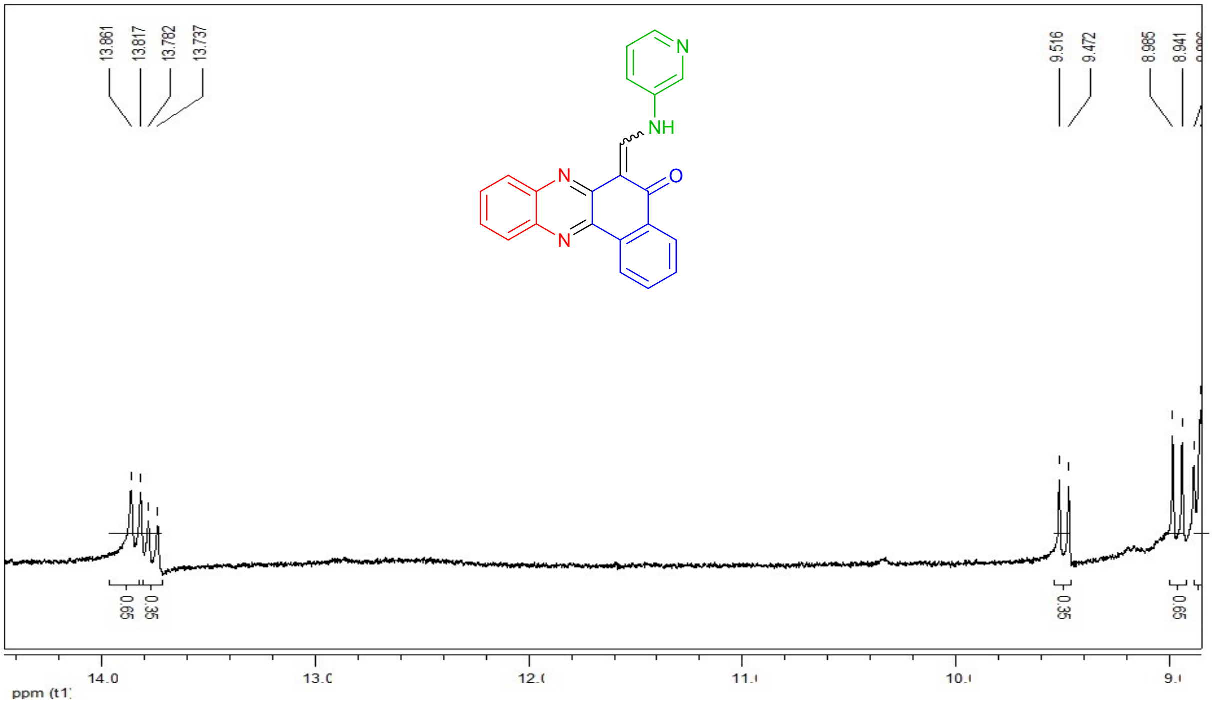

^{1}H-NMR spectrum of compound **6d** (300 MHz, DMSO-d_6)

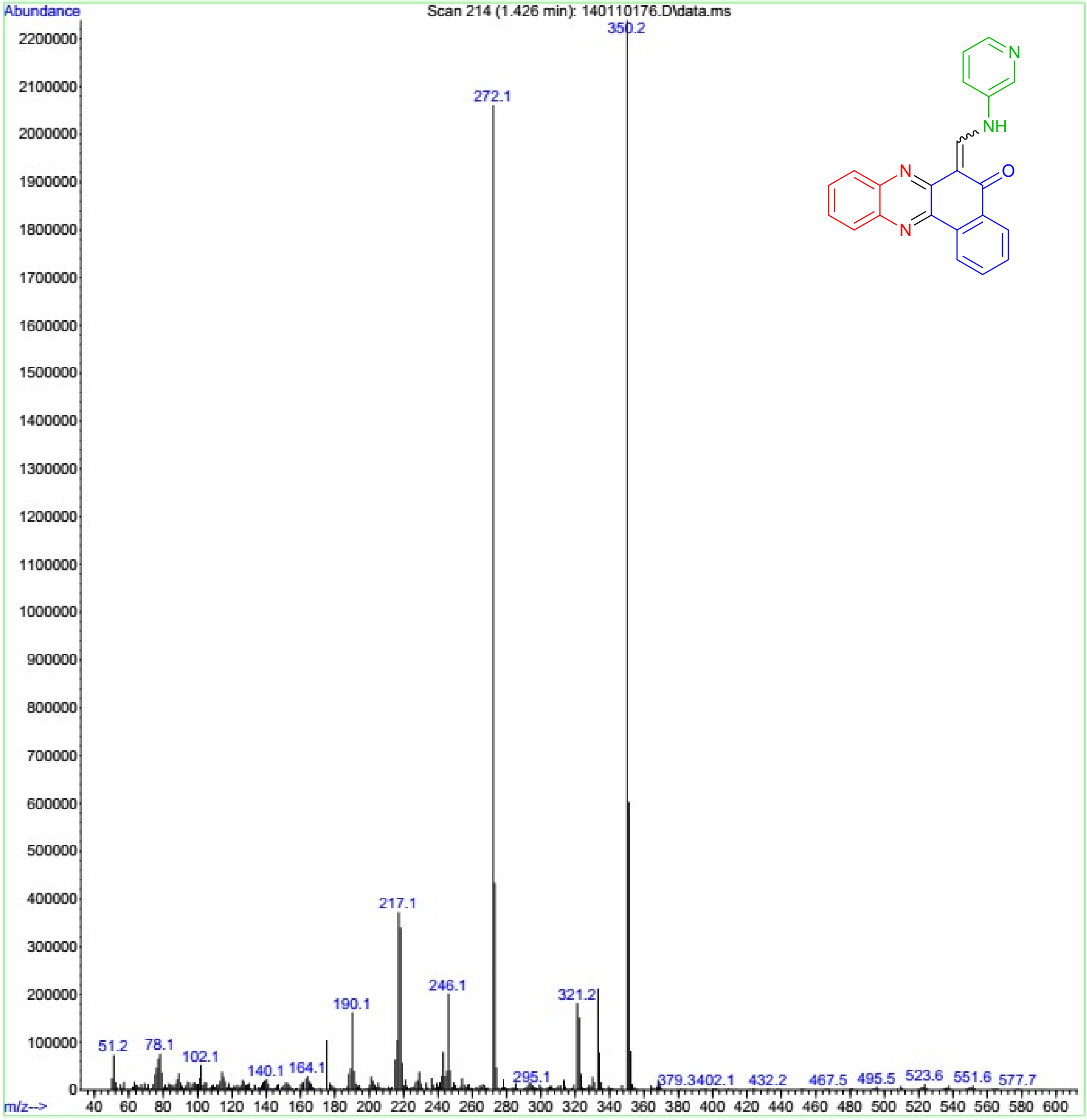

Mass spectrum of compound **6d**

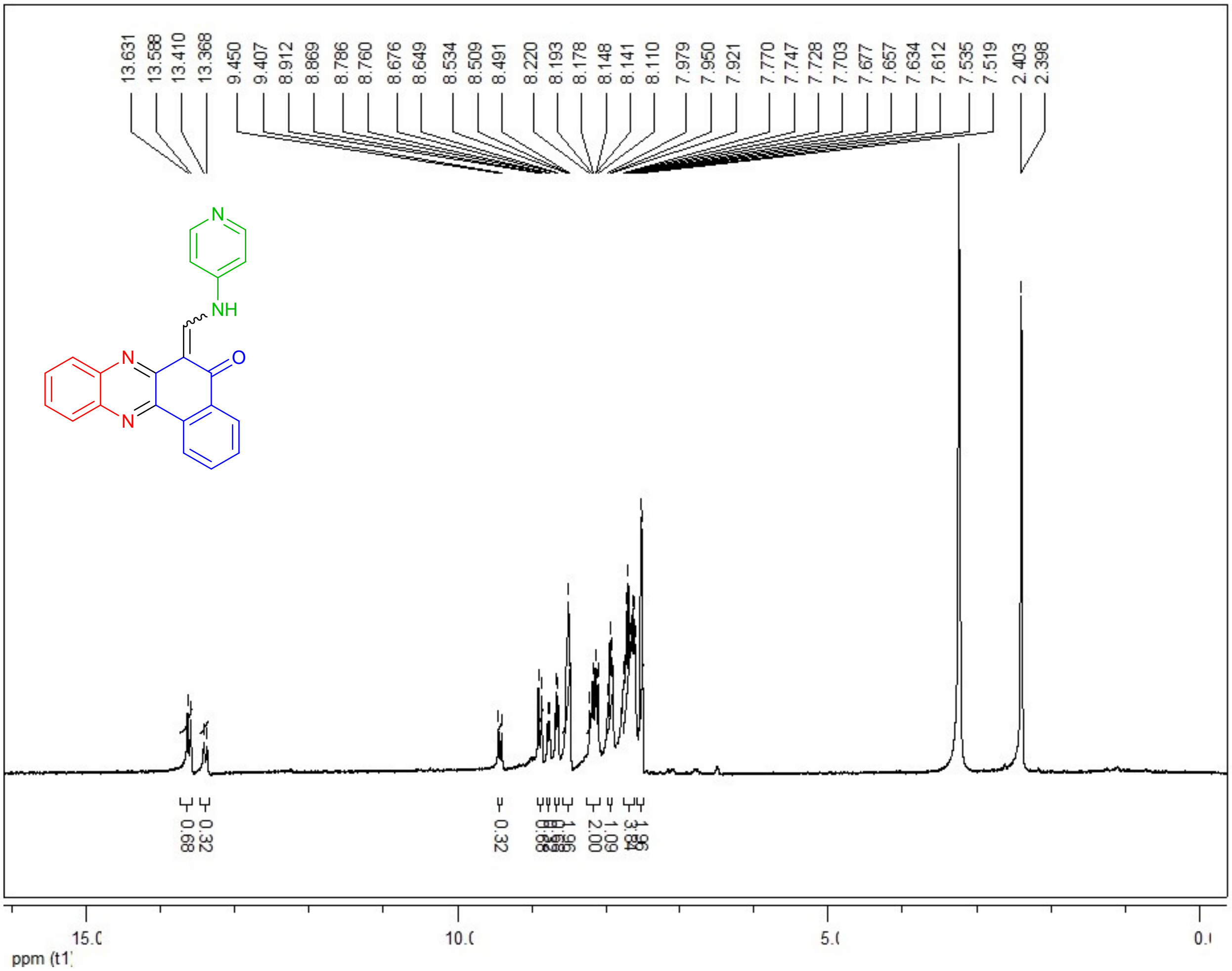

^{1}H-NMR spectrum of compound **6e** (300 MHz, DMSO-d_6)

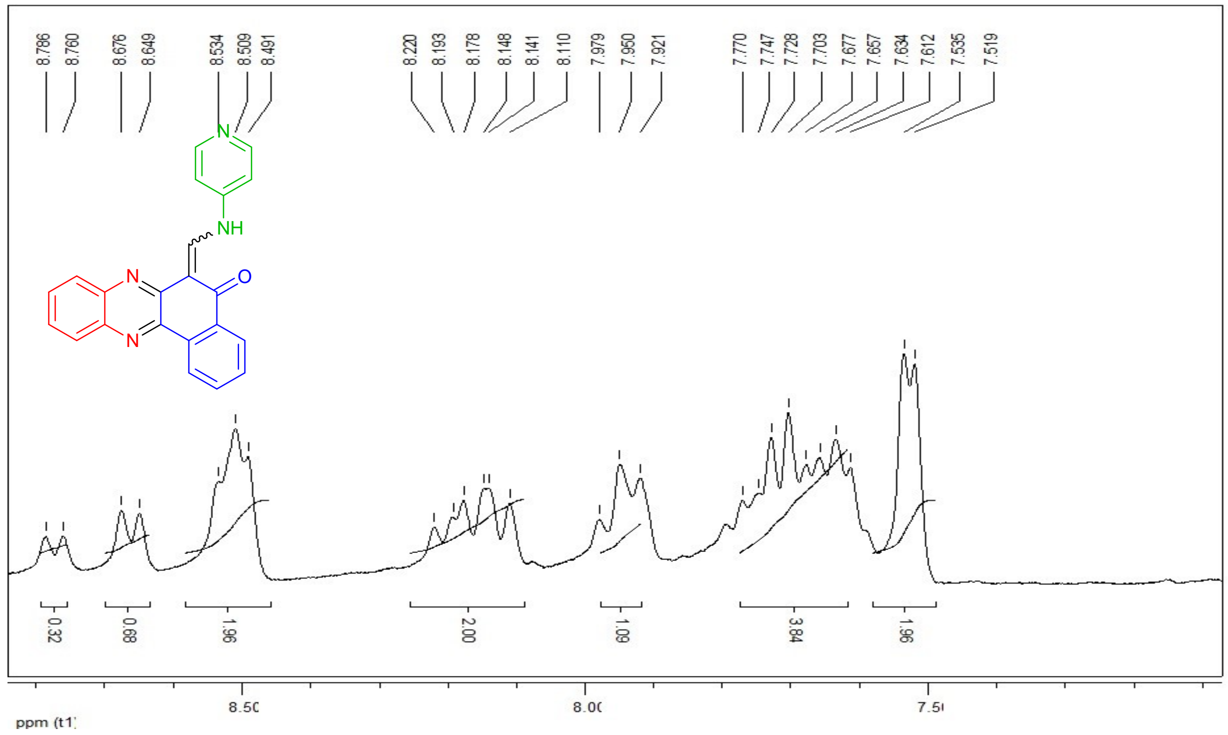

^{1}H-NMR spectrum of compound **6e** (300 MHz, DMSO-d_6)

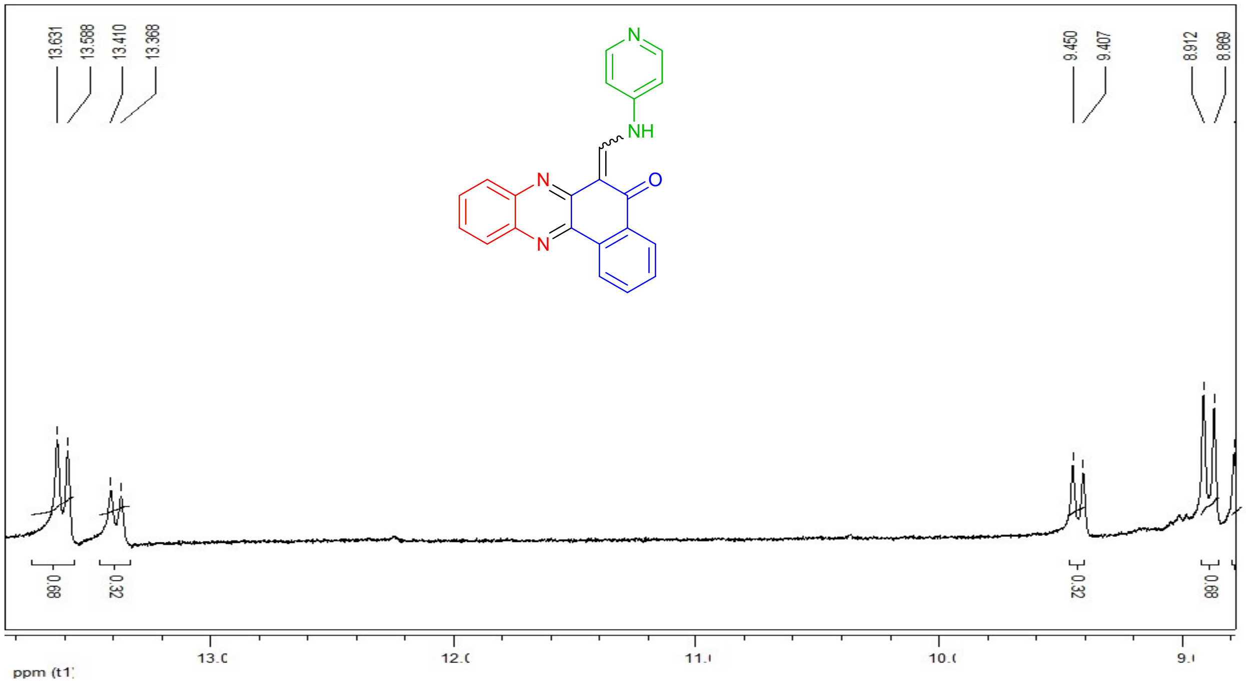

^{1}H-NMR spectrum of compound **6e** (300 MHz, DMSO-d_6)

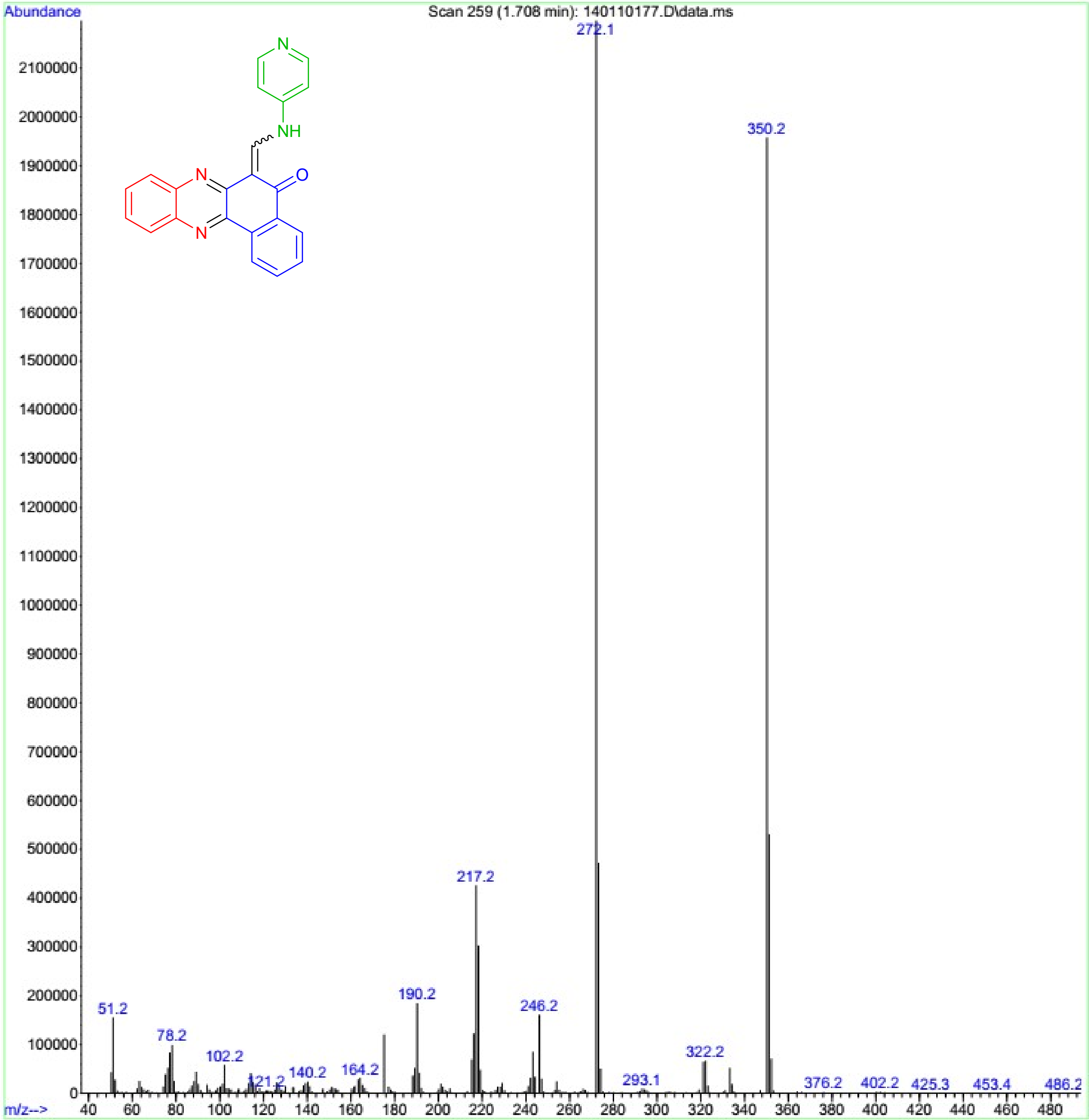

Mass spectrum of compound **6e**

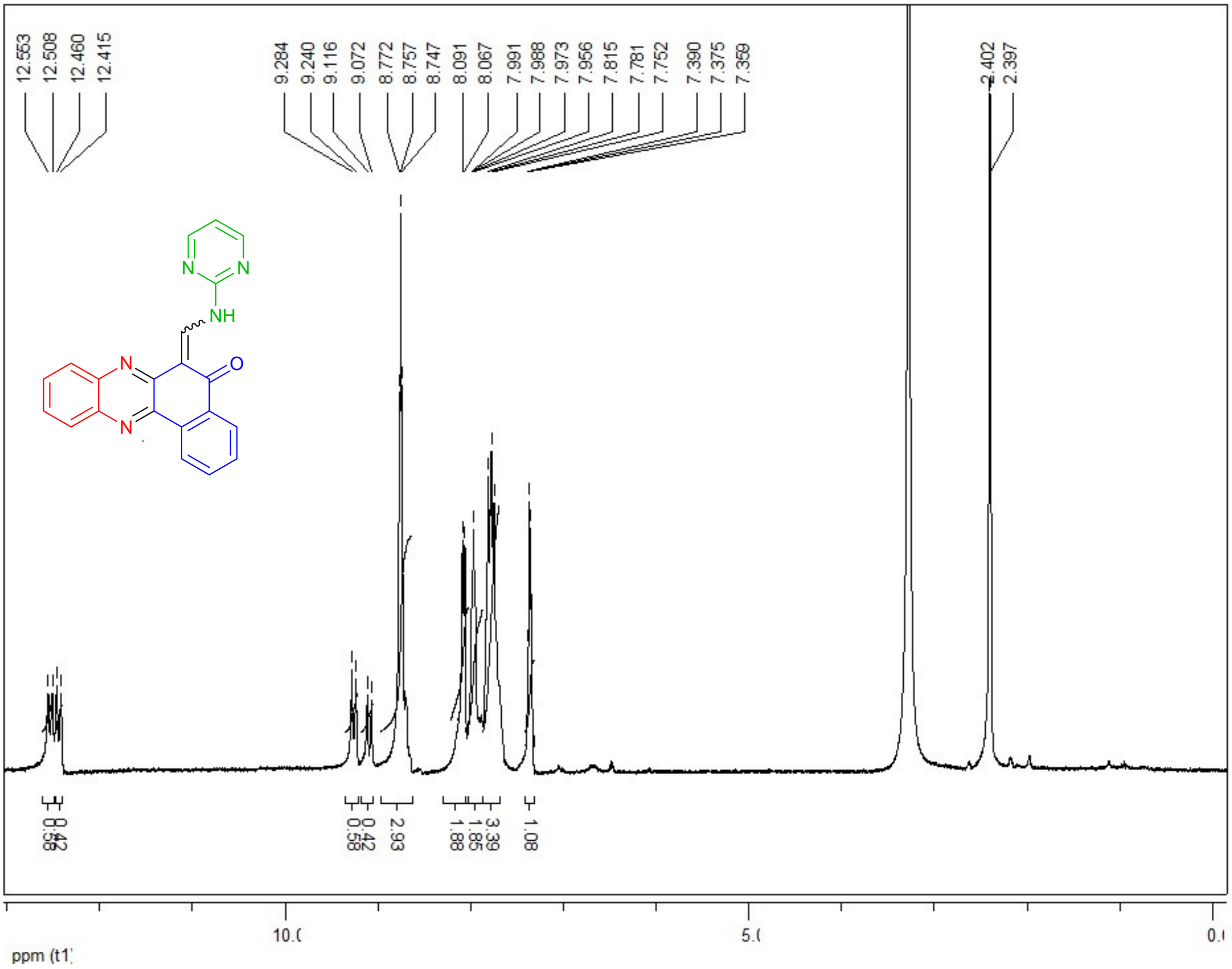

^{1}H-NMR spectrum of compound **6f** (300 MHz, DMSO-d_6)

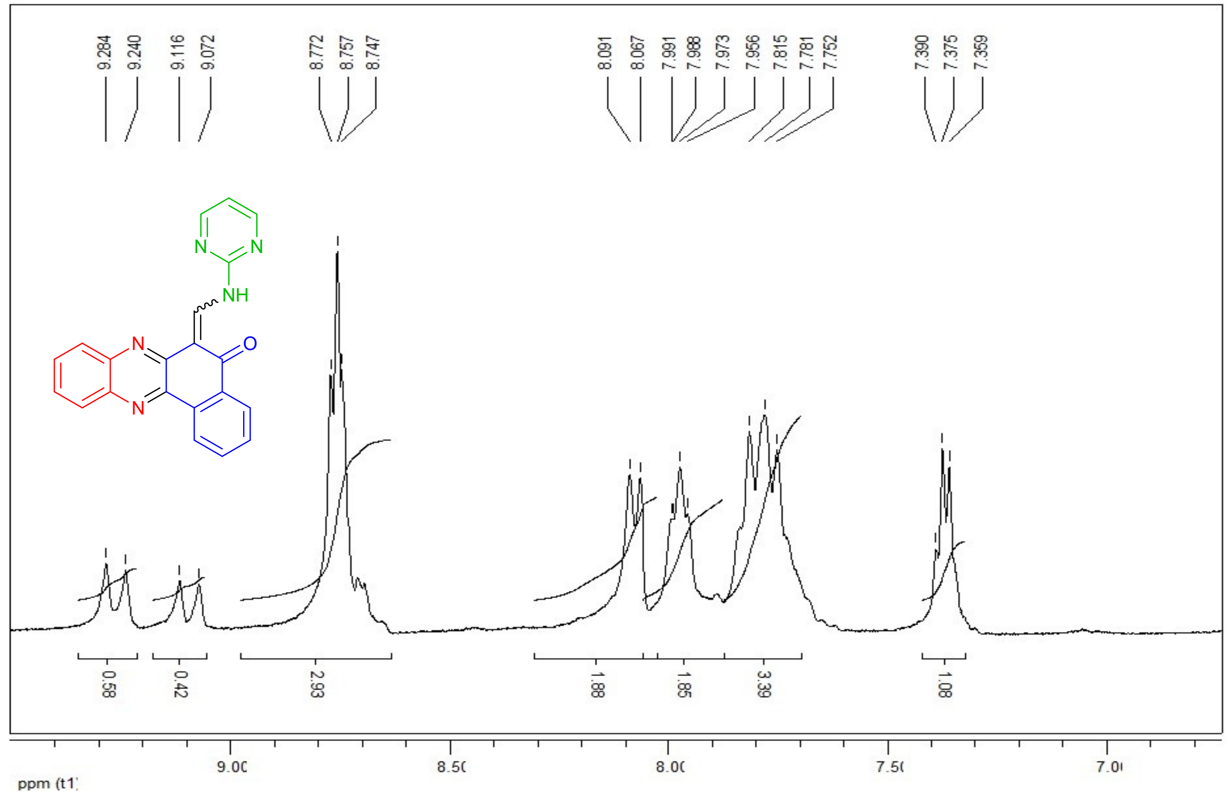

^{1}H-NMR spectrum of compound **6f** (300 MHz, DMSO-d_6)

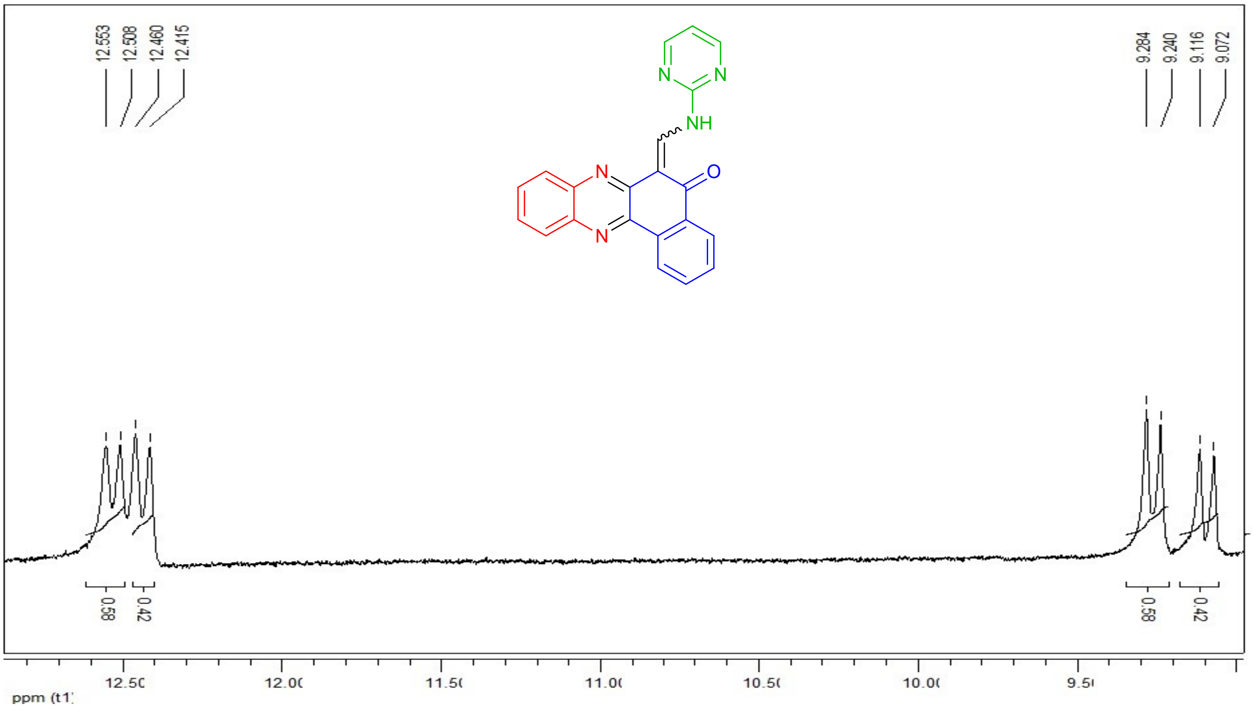

^{1}H-NMR spectrum of compound **6f** (300 MHz, DMSO-d_6)

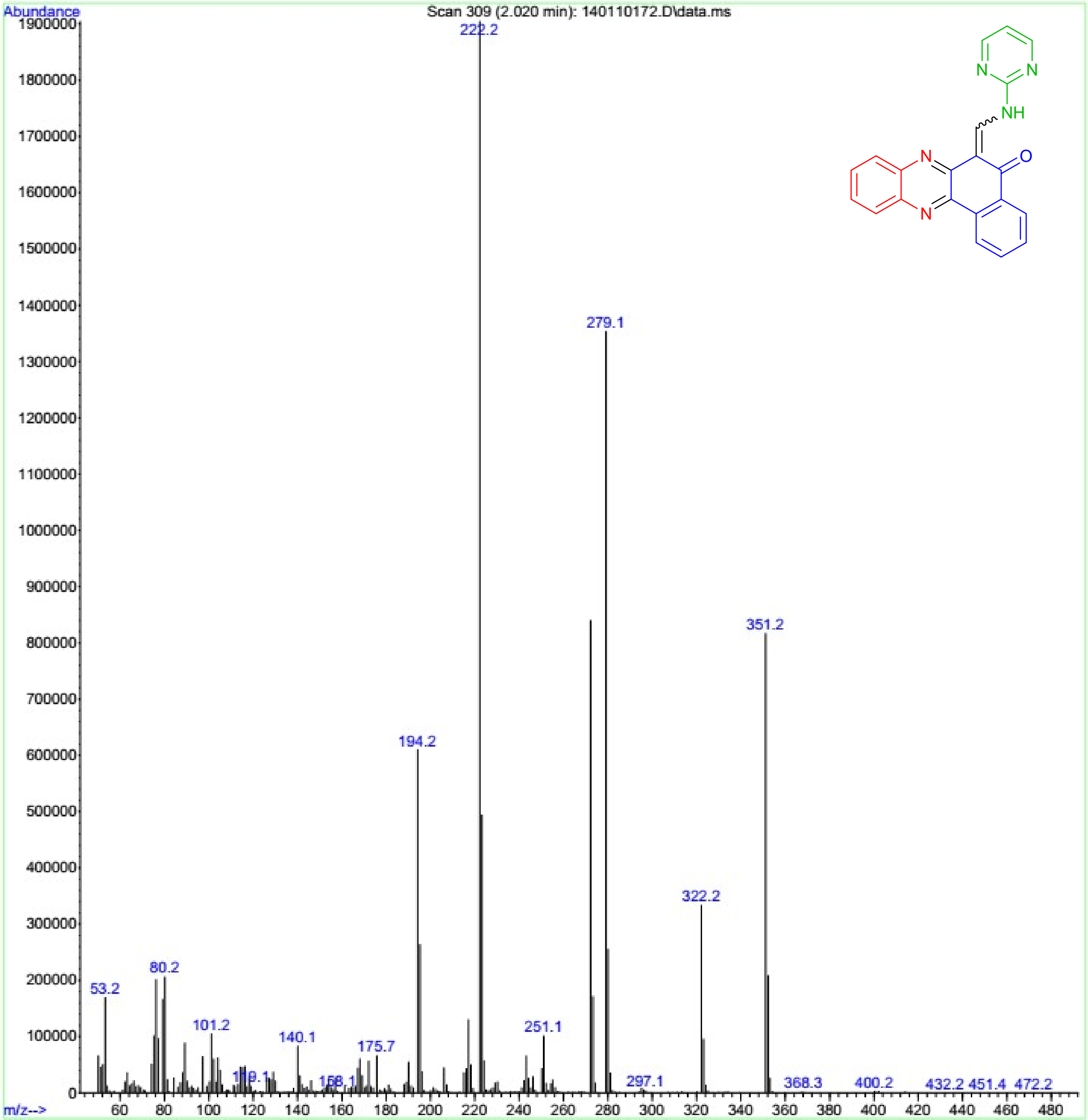

Mass spectrum of compound **6f**

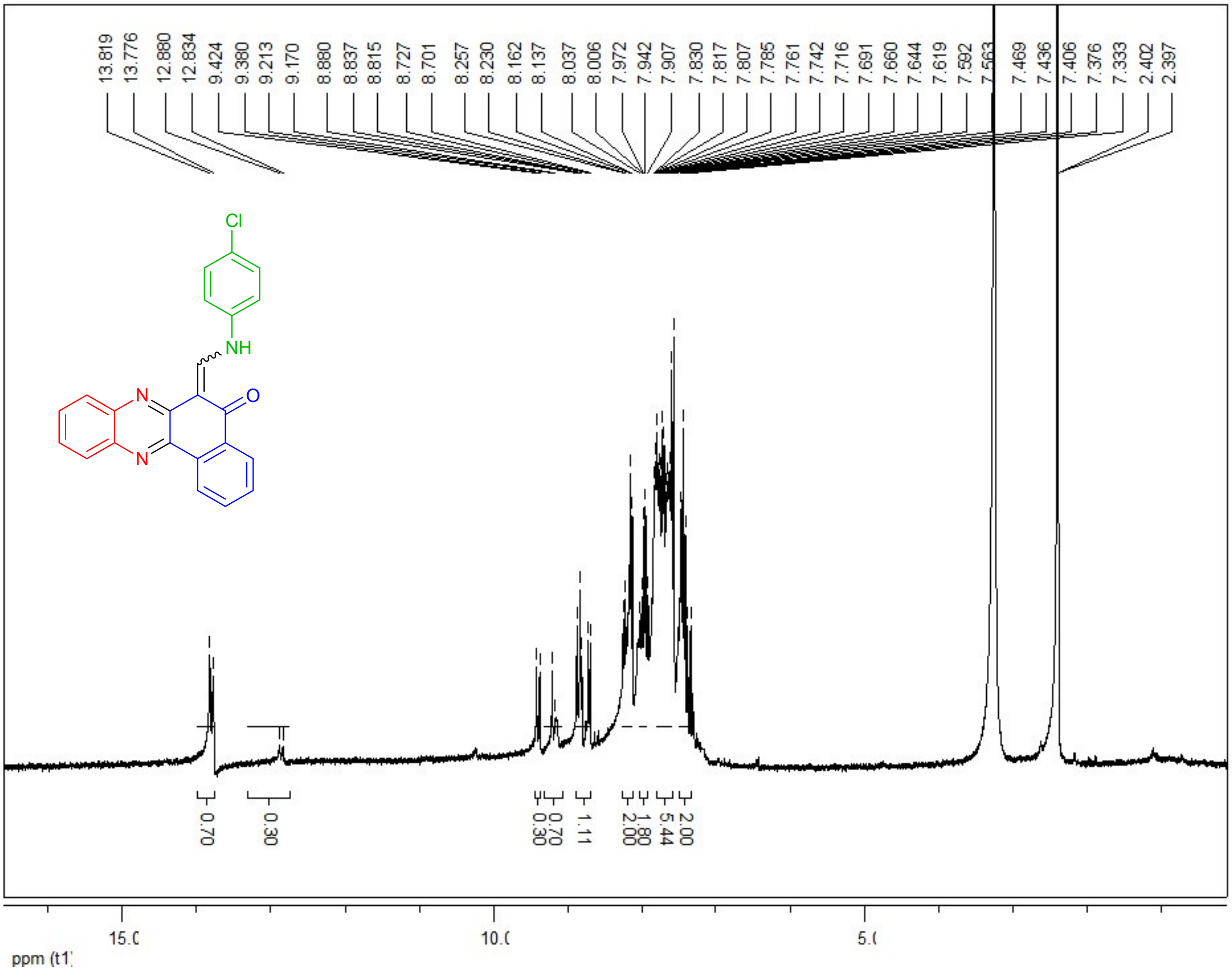

^{1}H-NMR spectrum of compound **6g** (300 MHz, DMSO-d_6)

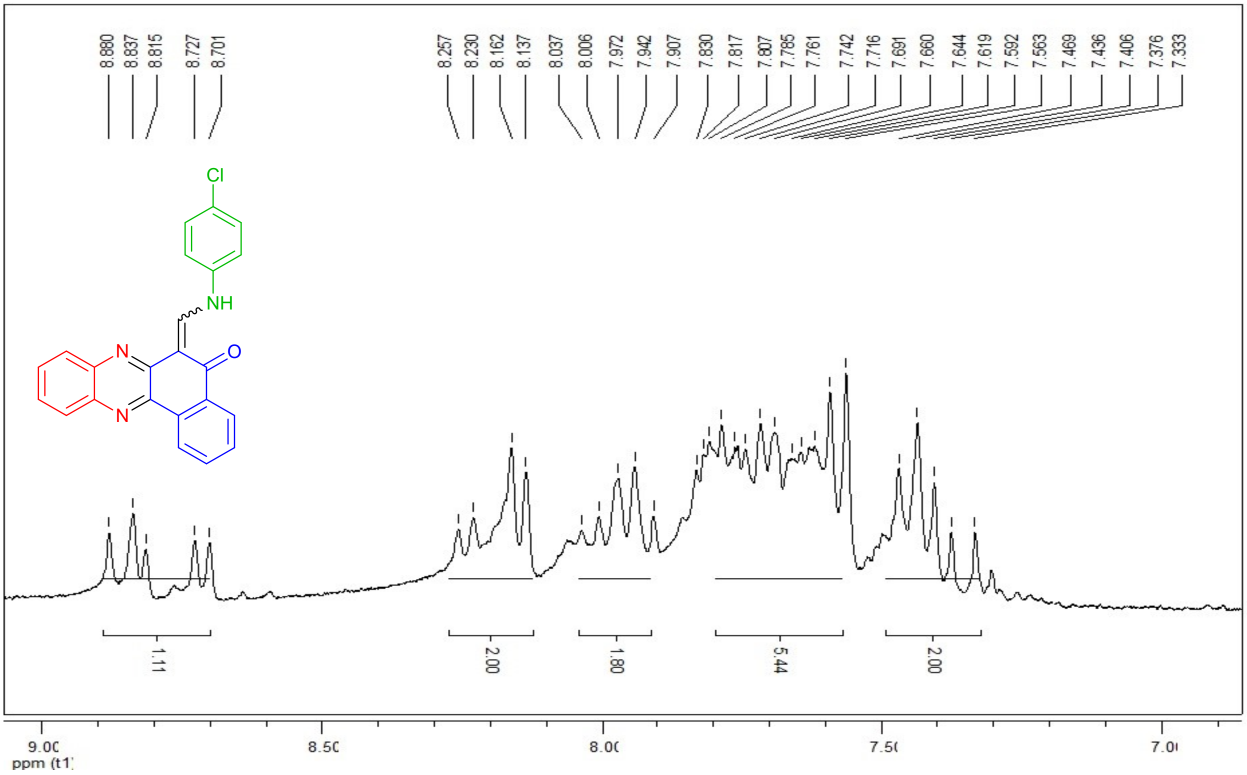

^{1}H-NMR spectrum of compound **6g** (300 MHz, DMSO-d_6)

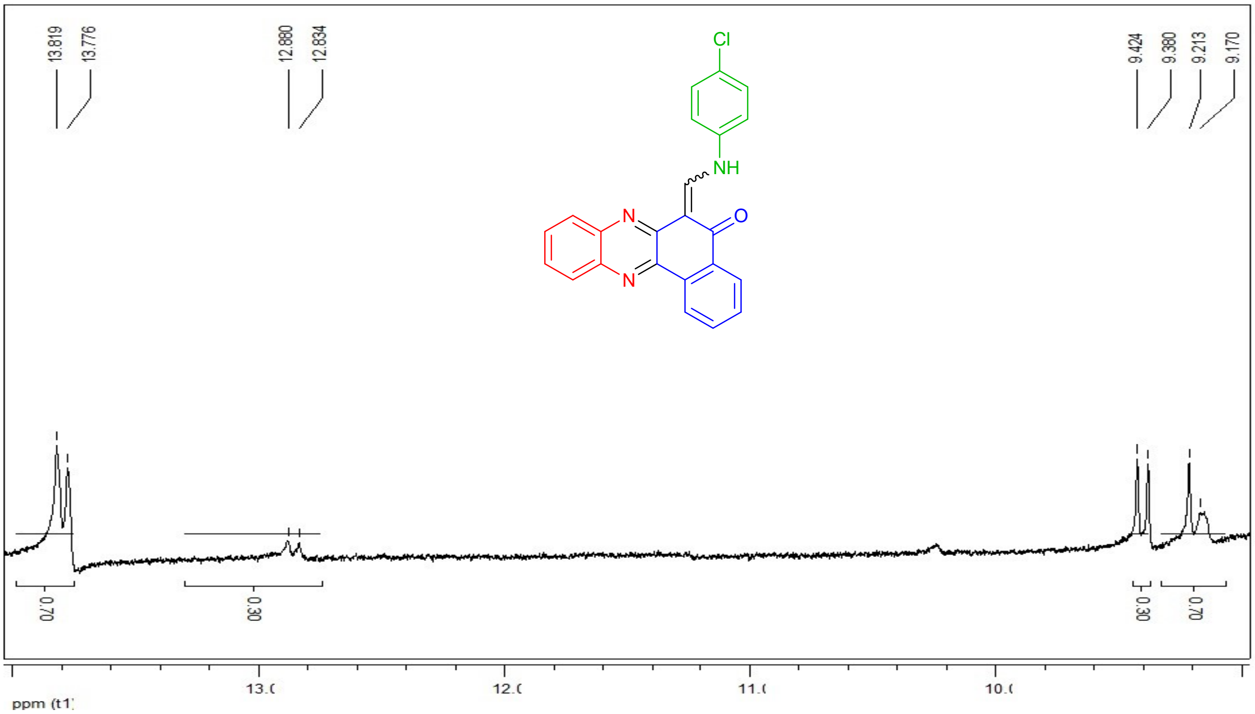

^{1}H-NMR spectrum of compound **6g** (300 MHz, DMSO-d_6)

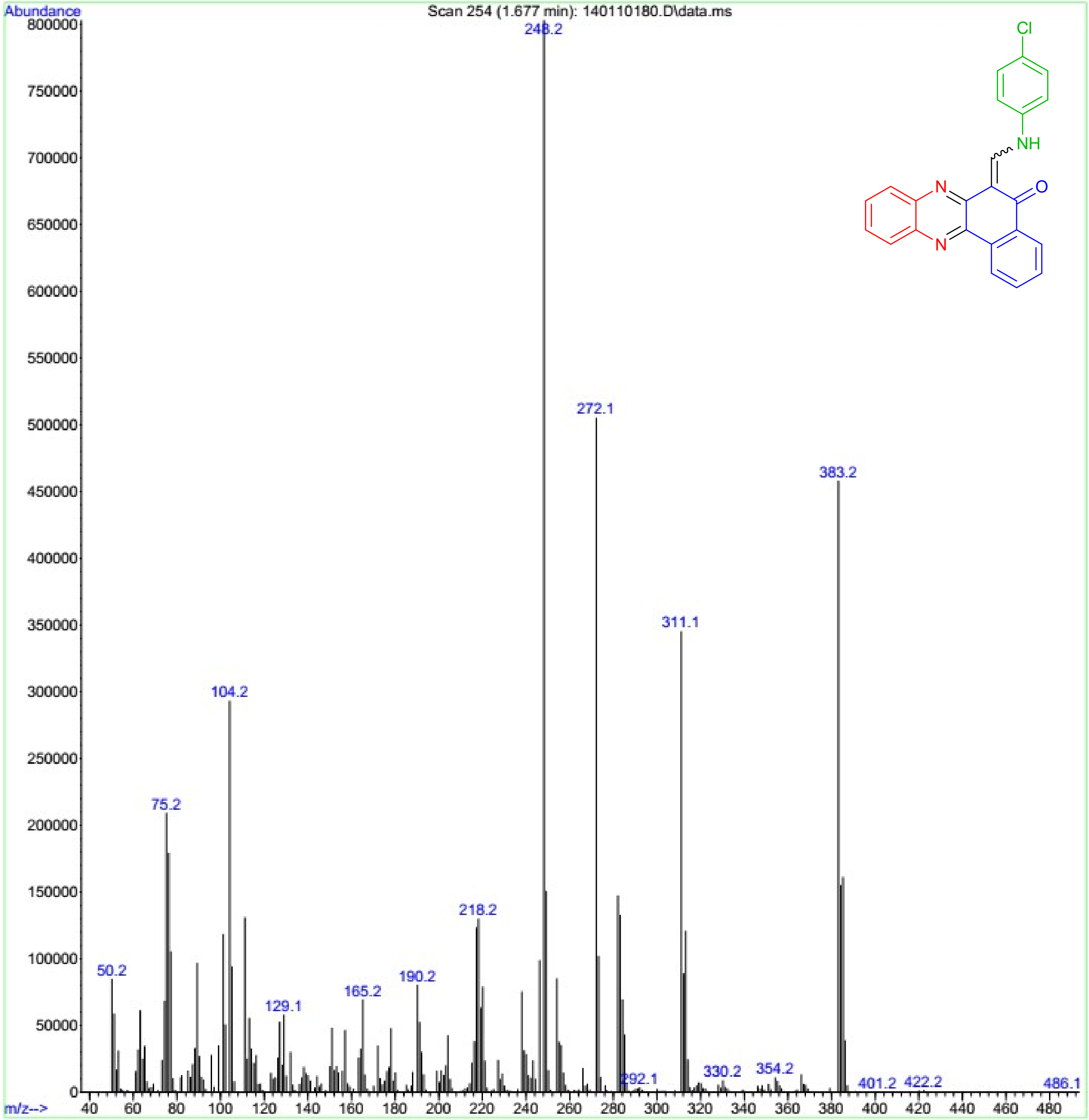

Mass spectrum of compound **6g**

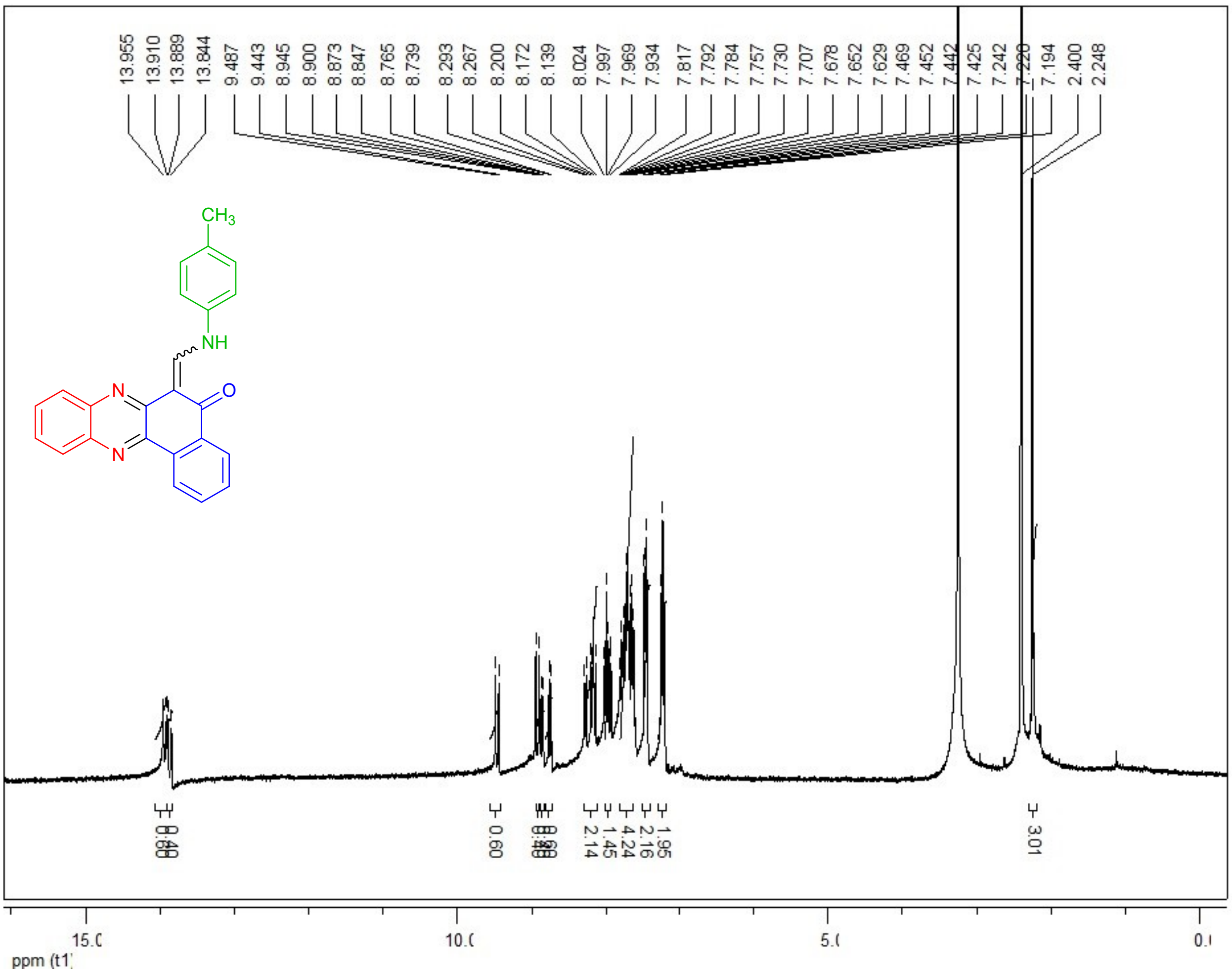

^{1}H-NMR spectrum of compound **6h** (300 MHz, DMSO-d_6)

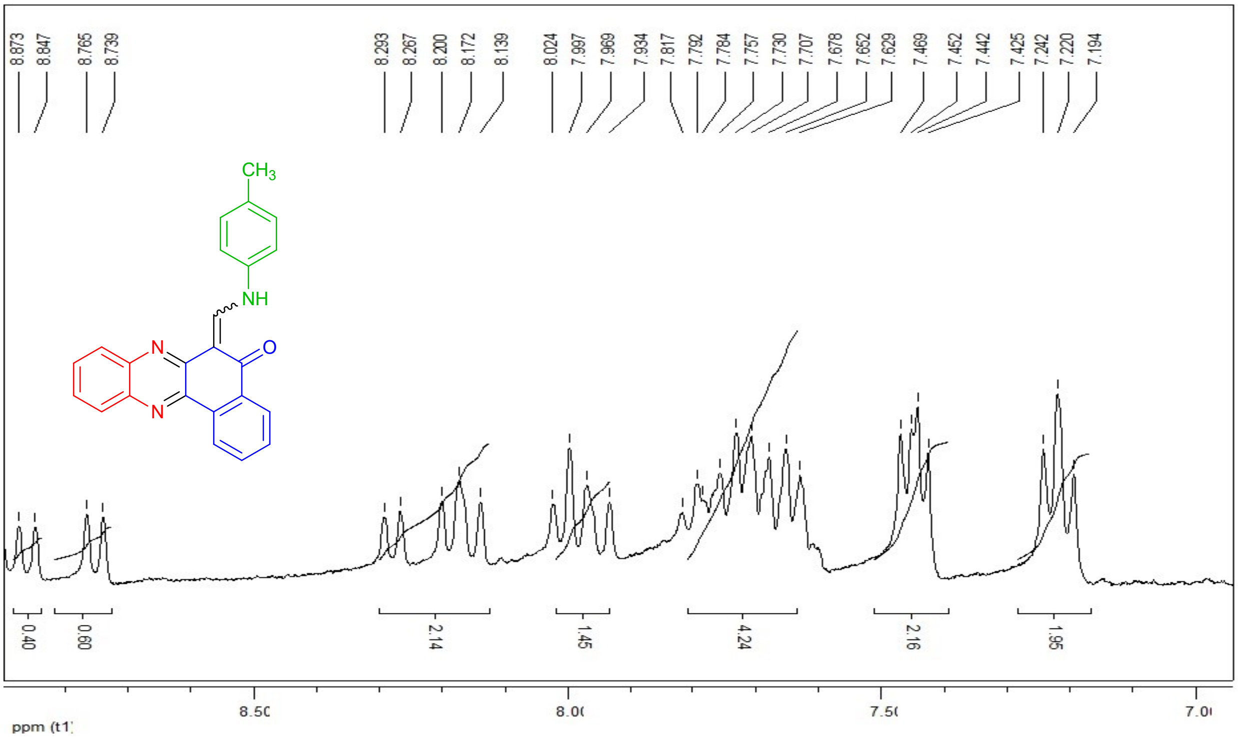

^{1}H-NMR spectrum of compound **6h** (300 MHz, DMSO-d_6)

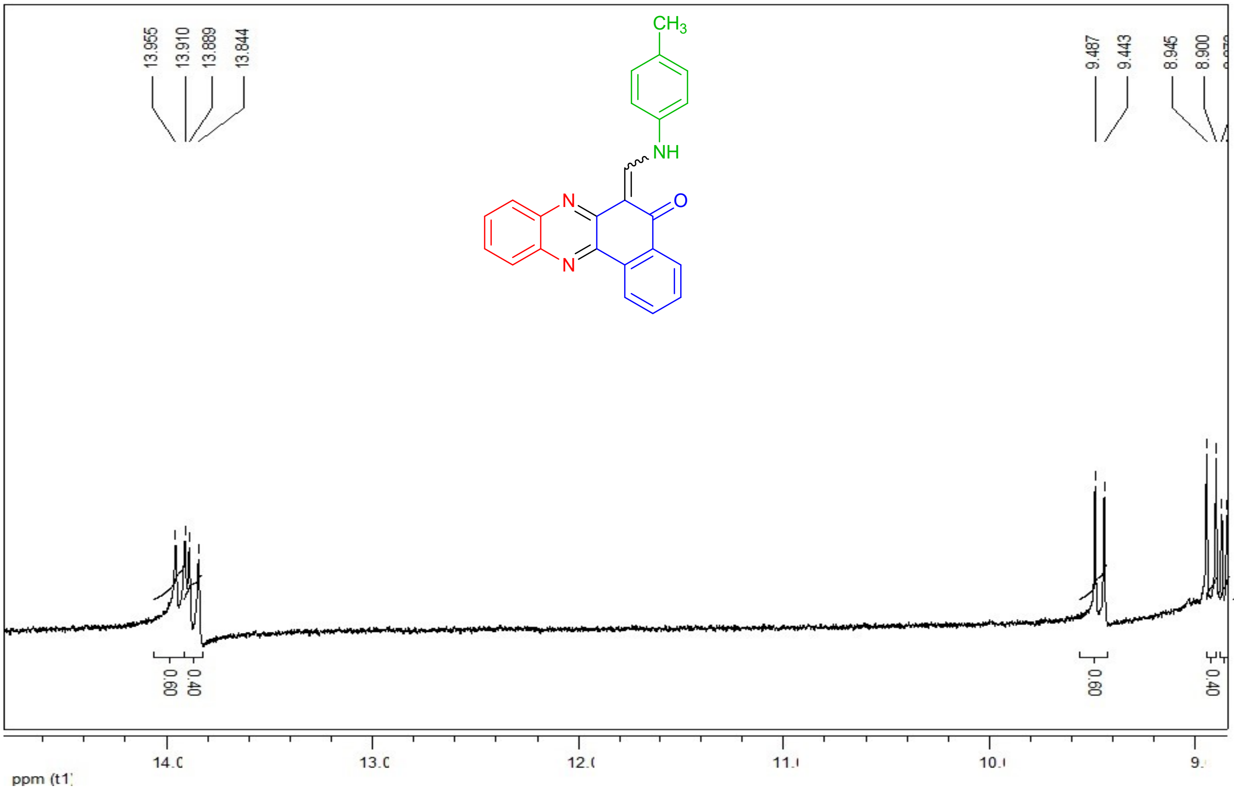

^{1}H-NMR spectrum of compound **6h** (300 MHz, DMSO-d_6)

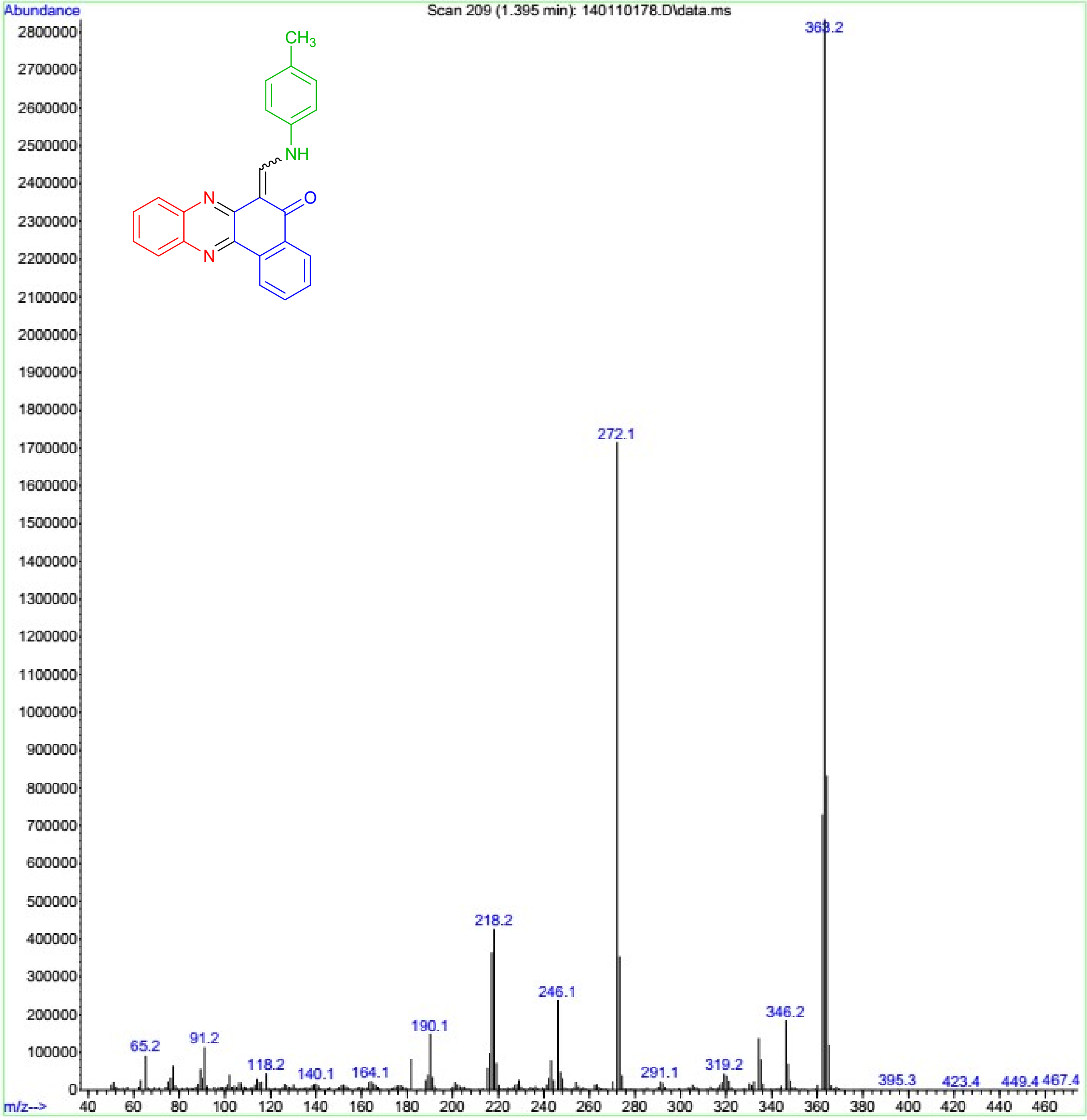

Mass spectrum of compound **6h**

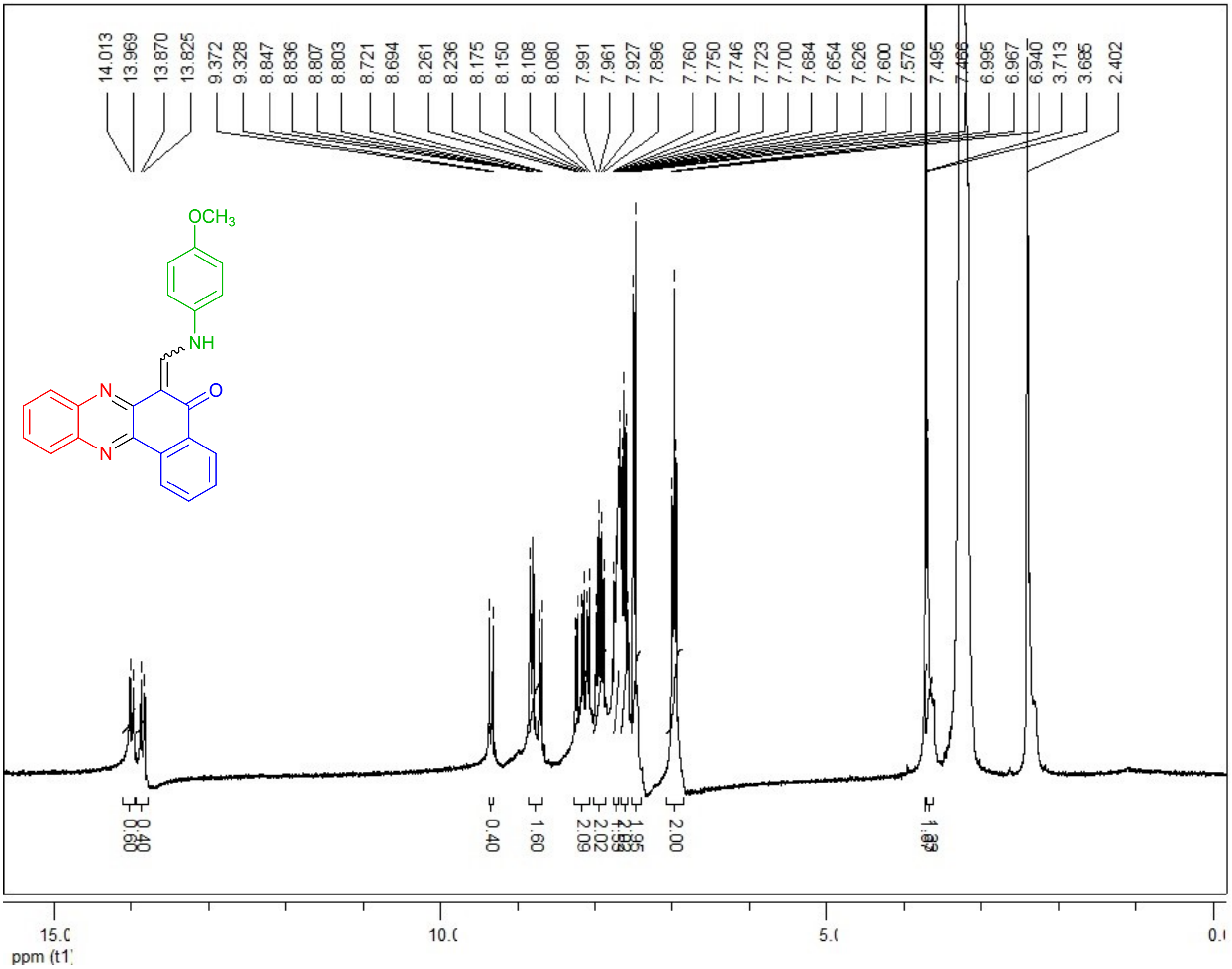

^{1}H-NMR spectrum of compound **6i** (300 MHz, DMSO-d_6)

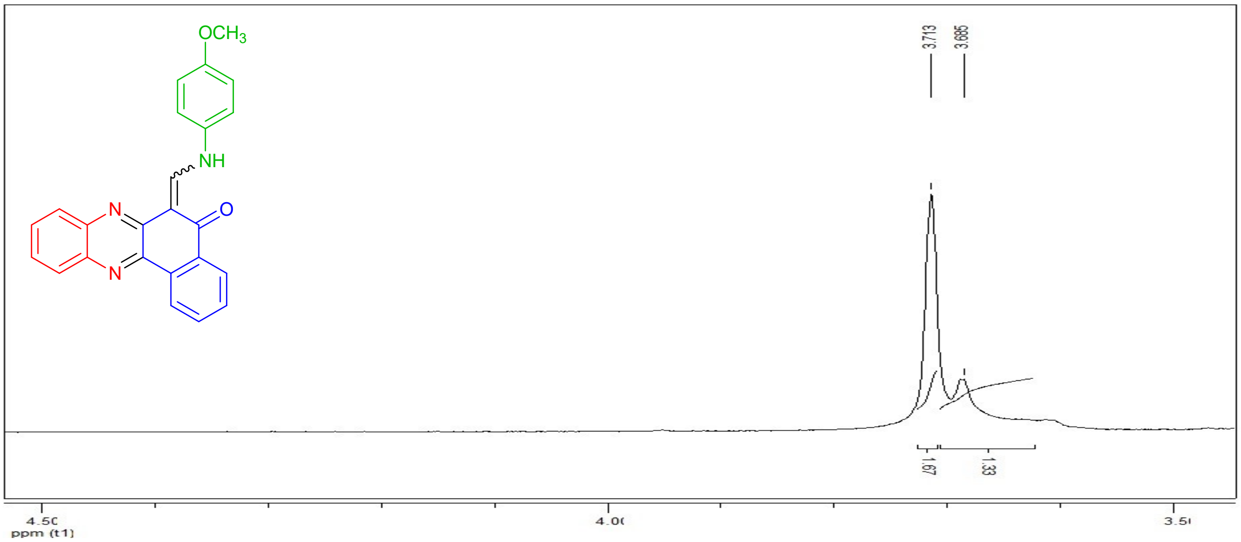

^{1}H-NMR spectrum of compound **6i** (300 MHz, DMSO-d_6)

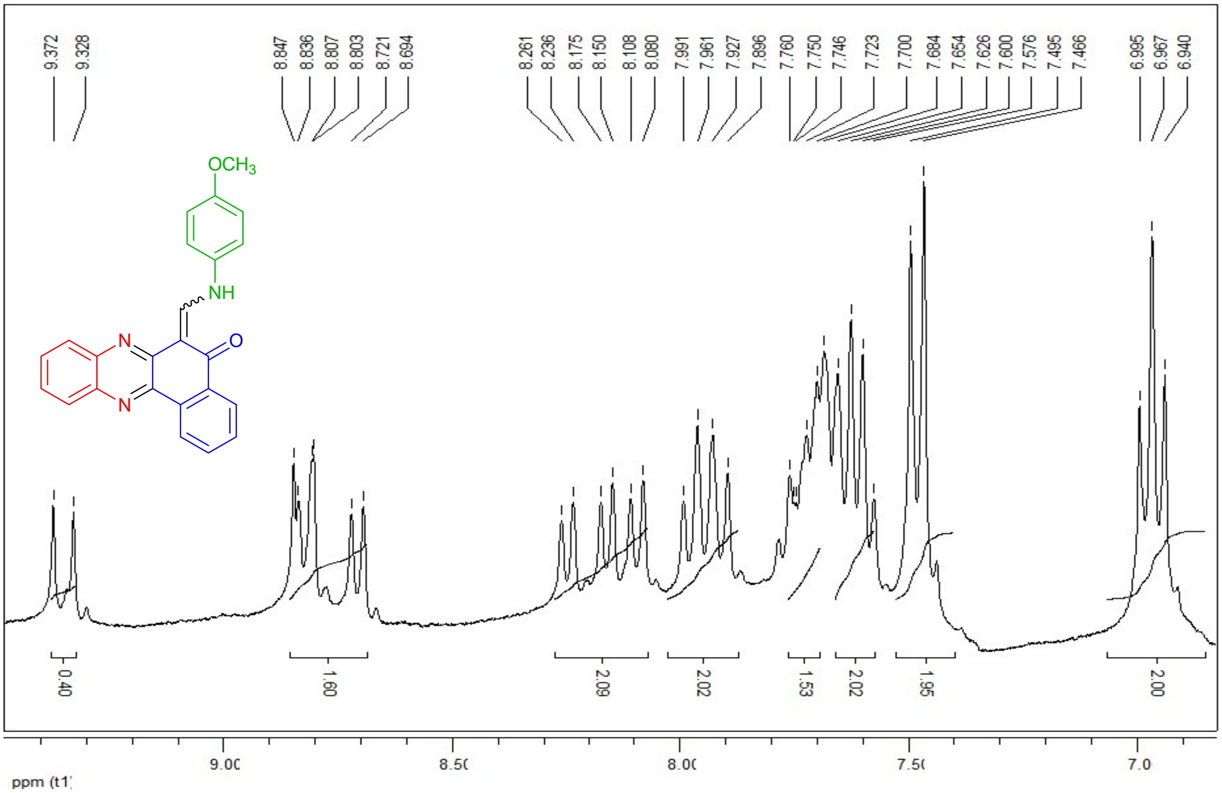

^{1}H-NMR spectrum of compound **6i** (300 MHz, DMSO-d_6)

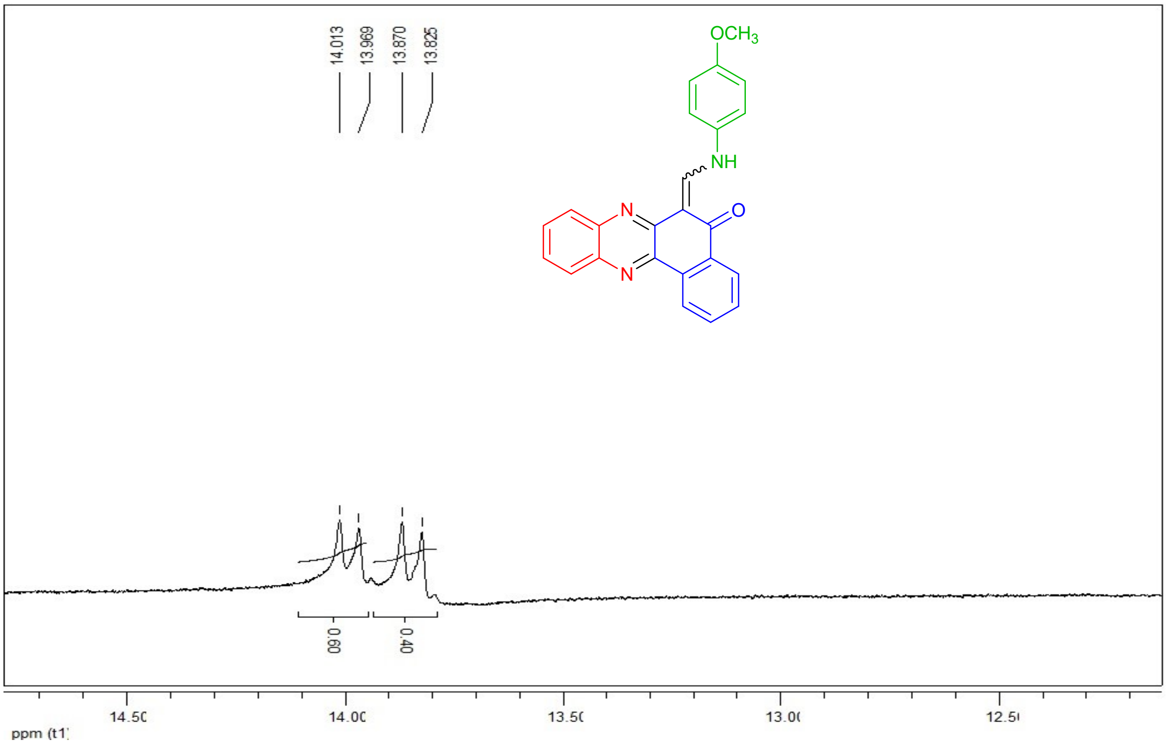

^{1}H-NMR spectrum of compound **6i** (300 MHz, DMSO-d_6)

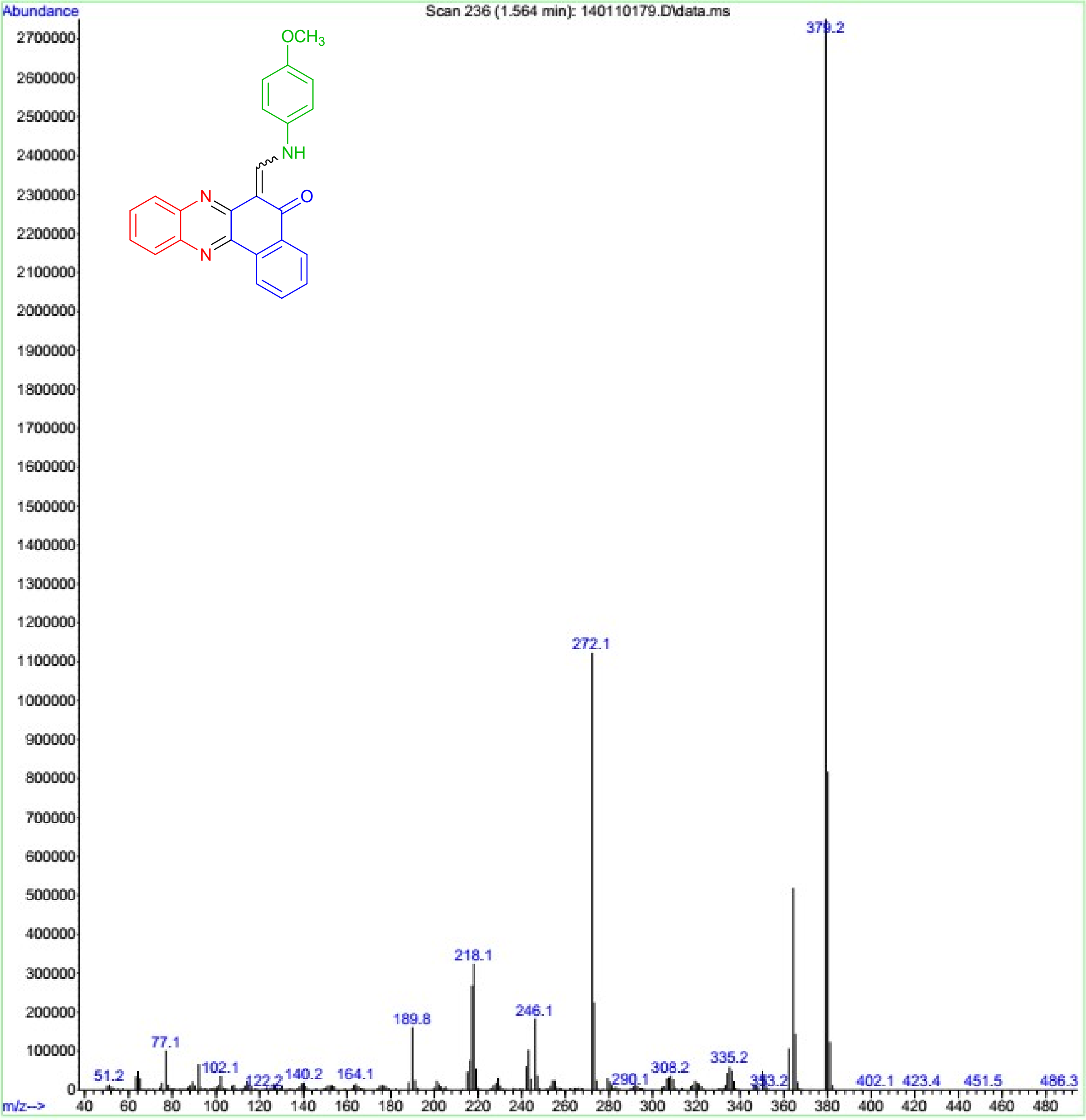

Mass spectrum of compound **6i**
